# Panhematopoietic RNA barcoding enables kinetic measurements of nucleate and anucleate lineages and the activation of myeloid clones following acute platelet depletion

**DOI:** 10.1186/s13059-023-02976-z

**Published:** 2023-06-27

**Authors:** Edyta E. Wojtowicz, Jayna J. Mistry, Vladimir Uzun, Charlotte Hellmich, Anita Scoones, Desmond W. Chin, Laura M. Kettyle, Francesca Grasso, Allegra M. Lord, David J. Wright, Graham J. Etherington, Petter S. Woll, Mirjam E. Belderbos, Kristian M. Bowles, Claus Nerlov, Wilfried Haerty, Leonid V. Bystrykh, Sten Eirik W. Jacobsen, Stuart A. Rushworth, Iain C. Macaulay

**Affiliations:** 1 Earlham Institute, Norwich Research Park, Norwich, UK; 2 Norwich Medical School, University of East Anglia, Norwich, UK; 3 Department of Cell and Molecular Biology, Karolinska Institutet, Stockholm, Sweden; 4 Department of Medicine, Huddinge, Center for Hematology and Regenerative Medicine, Karolinska Institutet, Karolinska University Hospital, Stockholm, Sweden; 5 Norfolk and Norwich University Hospital, Norwich, UK; 6 Princess Maxima Center for Pediatric Oncology, Utrecht, The Netherlands; 7 MRC Molecular Haematology Unit, MRC Weatherall Institute of Molecular Medicine, University of Oxford, Oxford, UK; 8 School of Biological Sciences, University of East Anglia, Norwich, UK; 9 European Research Institute for the Biology of Ageing (ERIBA), University Medical Center of Groningen (UMCG), University of Groningen, Groningen, The Netherlands

**Keywords:** Lineage tracing, Platelets, Platelet depletion, Clonal switching, Hematopoiesis

## Abstract

**Background:**

Platelets and erythrocytes constitute over 95% of all hematopoietic stem cell output. However, the clonal dynamics of HSC contribution to these lineages remains largely unexplored.

**Results:**

We use lentiviral genetic labeling of mouse hematopoietic stem cells to quantify output from all lineages, nucleate, and anucleate, simultaneously linking these with stem and progenitor cell transcriptomic phenotypes using single-cell RNA-sequencing. We observe dynamic shifts of clonal behaviors through time in same-animal peripheral blood and demonstrate that acute platelet depletion shifts the output of multipotent hematopoietic stem cells to the exclusive production of platelets. Additionally, we observe the emergence of new myeloid-biased clones, which support short- and long-term production of blood cells.

**Conclusions:**

Our approach enables kinetic studies of multi-lineage output in the peripheral blood and transcriptional heterogeneity of individual hematopoietic stem cells. Our results give a unique insight into hematopoietic stem cell reactivation upon platelet depletion and of clonal dynamics in both steady state and under stress.

**Supplementary Information:**

The online version contains supplementary material available at 10.1186/s13059-023-02976-z.

## Background

Long-term hematopoietic stem cells (LT-HSCs) are rare bone marrow (BM)-resident multipotent cells with the potential to self-renew and to replenish all nucleated [1] and anucleated peripheral blood (PB) cells [2], sustaining long-term multilineage reconstitution after physiological and clinical challenges including chemotherapy and bone marrow transplantation [3, 4]. Allogeneic LT-HSC transplantation remains the only curative treatment modality for numerous hematological malignancies; however, inefficient blood lineage replenishment, especially neutropenia and thrombocytopenia, remains a major cause of morbidity and mortality [5, 6]. Single-cell transplantation experiments have uncovered significant heterogeneity among reconstituting LT-HSCs, which may reflect different propensities for lineage commitment by distinct myeloid-, lymphoid-, and platelet-biased LT-HSCs [2, 7–10]. Given such a high level of heterogeneity, it is unclear how different LT-HSCs coexist in a polyclonal BM microenvironment and how many stem cell clones are simultaneously actively supporting the production of nucleated and anucleated cells. Furthermore, although recent reports have shown that platelet-biased LT-HSCs may represent a fast-track route to platelet production [11, 12], the response of individual LT-HSCs and their clonal progeny to acute depletion of the platelet lineage and the transcriptional landscape underlying these clonal changes remains unexplored.

The quantitative clonal analysis depends on the labeling and detection methods used. Cellular barcoding methods using genomic DNA were recently implemented for the clonal analysis of LT-HSCs [13–15]. This approach provided detailed information about LT-HSC subsets and clonal kinetics in myeloid and lymphoid lineages during hematopoiesis after transplantation [14, 16] or aging [17]. Recently, RNA-based barcoding has been applied in studies of the nucleated hematopoietic cells in the BM; however, the labeling protocol and cell isolation method applied did not effectively label or enrich platelets and erythrocytes [18]. Fan et al. performed a clonal analysis of the erythroid lineage in macaque, including anucleate erythrocytes in the PB upon transplantation [19]. However, platelets, in spite of their clinical significance, abundance (up to 60% of HSC output [2], and the clear indications that their progenitors, megakaryocytes (MKs), are closely linked to HSCs [20], have so far been omitted from the clonal analysis. Platelet biogenesis is completely distinct from erythrocytes and all other blood lineages: mature platelets are cell fragments generated through cytoplasmic fragmentation of MKs [21]. On average, each MK is thought to be able to produce 3000–5000 platelets [22], and it is thought that MKs themselves exhibit heterogeneous phenotypes in terms of platelet output and function [23]. The clonal composition and kinetics within this lineage therefore represent a unique yet unexplored aspect of hematopoietic biology.

Here, we present the barcoding of all nucleated and anucleated PB lineages (including platelets) for kinetic clonal studies of PB reconstitution. We applied an RNA cellular barcoding method to FACS-purified LT-HSCs in combination with single-cell RNA sequencing (scRNA-seq) to perform kinetic clonal tracking of nucleated and anucleated blood cell populations in the hematopoietic system. These data sets were then compared with molecular signatures of LT-HSC, platelet (MkP), and erythroid (CFU-E) progenitors. We have taken advantage of a lentiviral barcoding library, where the barcode is located in the 3′ end of an eGFP transcript and is therefore present in both the genome of the cells and expressed as mRNA [24]. This approach is versatile, sensitive, and applicable to other model systems including human cord blood, bone marrow cells, or primary leukemic cells [24].

Through cellular barcoding and transplantation, we have characterized the clone size and repopulation potential in 596 LT-HSCs. Our data provide detailed insight into the functional differences and the dynamics of LT-HSC response to stress. We demonstrate that most transplanted clones consistently contribute to hematopoiesis and have multipotent potential to produce myeloid, lymphoid, erythroid, and platelet progeny, supporting the clonal stability model of hematopoiesis. Importantly, upon acute platelet depletion, we observe the most striking changes in the myeloid lineage: (1) a reprogramming of multipotent clones, which start to exclusively produce platelets, and (2) the emergence of new clones producing only myeloid progeny to compensate for the reduced myeloid output of reprogrammed, multipotent clones. In the long term, the clones activated to produce myeloid cells show functional diversity. The majority of clones remain multipotent and contribute to myeloid, erythroid, and B cells, while platelet output is supported by newly recruited platelet clones. Others have a peak output at 10 days after the platelet depletion and then support myeloid lineage in the long term. Our single-cell RNA-seq analysis revealed that genes differentially expressed between reprogrammed and multipotent clones are involved in DNA replication and repair, cell cycle, and methylation. These results have potential clinical implications for thrombocytopenia in patients suffering from immune thrombocytopenia purpura [25], undergoing chemotherapy or BM transplantation through quantifying the frequency of repopulating LT-HSCs and unraveling how the system returns to the steady state upon acute platelet depletion.

## Results

### RNA barcoding combined with high-throughput sequencing enables quantitative clonal analysis

**Fig. 1:**
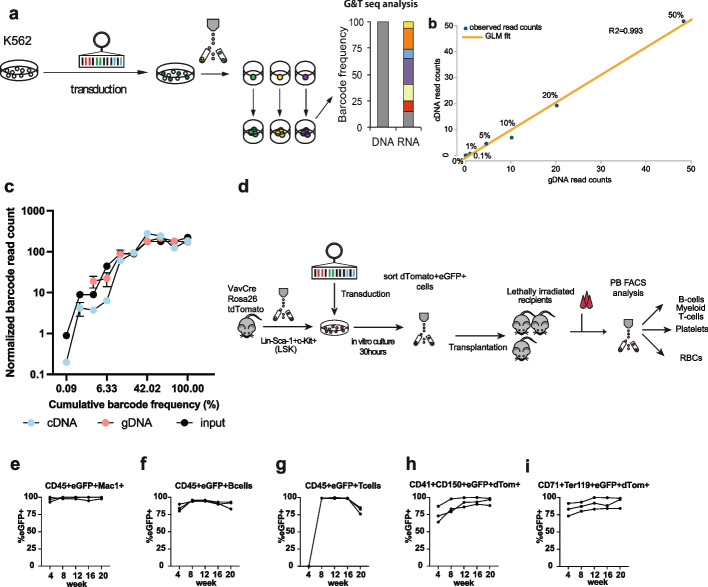
In vitro and in vivo validation of RNA barcoding approach for stable, long-term clonal kinetics studies of hematopoiesis. **A** Experimental setup for in vitro validation of quantitative RNA-based clonal studies. The K562 cell line was used for the transduction, and single eGFP + cells were FACS-sorted and expanded to generate monoclonal calibration samples. Four clones were used to create samples, where cells carrying different barcodes were mixed in known ratios-spiked in clone contributing 0.1%, 1%, 5%, 10%, 20%, or 50% of the total mix. **B** Generalized linear model (GLM) correlation between the read count for barcoded transcript retrieved in calibration samples from cDNA or gDNA. **C** Detection of unequally mixed barcodes in cDNA and gDNA samples, ten barcoded clones of the BaF3 cell line were mixed in 1:10:10:50:100:100:200:200:200:250 ratios, the smallest clone contributing at 0.089%. Presented are results from 2 technical replicates. **D** Experimental setup to test the silencing of eGFP in VavCre x Rosa26tdTomato LSK cells. **E**–**I** eGFP + chimerism changes in tdTomato + anucleate cells and CD45.2 + (donor-derived) nucleated cells. To calculate the silencing in specific lineage, we subtracted eGFP + cells from CD45.2 + cells (nucleated cells-CD19 + , Mac1 + or CD4/8 + cells) or from tdTomato + cells (all donor-derived anucleate cells); shown are the results from a single experiment in 3 recipient mice

Before in vivo analysis of clonal composition in anucleate and nucleated cells, we validated the sensitivity and accuracy of RNA and DNA barcoding assay in vitro using the K562 cell line as a model. DNA served as a positive control, being a widely accepted approach for in vivo lineage tracing [15, 16, 26]. A previously published lentiviral barcoding library [24] was used to transduce K562 cells. Individual barcoded cells were single-cell FACS purified before being propagated (Fig. 1A). Samples to test the performance of RNA barcoding were created by diluting between 1 and 5000 or 1 and 1120 cells with the same barcode with cells containing different barcodes. Using G&T-seq, we simultaneously isolated RNA and DNA from the same sample [27]. Individual samples were amplified with primers containing a unique multiplex tag and pooled multiplexed PCR products were sequenced (Fig. 1A). The sequencing results were analyzed using a previously published protocol [28] (the “Methods” section). Read counts for the barcodes in cDNA and gDNA samples were highly correlated between barcode abundance at different dilutions (*R* ^2^ = 0.993) using a generalized linear model fit (Fig. 1B). Comparison of cDNA and gDNA abundance using individual barcodes revealed consistently high correlation between both molecules (Pearson correlation *r* = 0.85, Additional file 1: Fig. S1A-B) that was higher compared to the previous study (*r* = 0.72) [18]. The analysis of highly unequally mixed samples allowed a reliable detection of clones that represent only 0.089% of the starting population, while they were missed at the DNA level (Fig. 1C). cDNA performed better at detecting smaller clones and showed similar sensitivity to gDNA when detecting the top 75% of all barcodes (Fig. 1C, Additional file 1: Fig. S1C, Additional file 2: Table S1A). These mixtures of cells are a better approximation of the clonal composition observed in vivo, where some clones are major contributors while others have low output. Thus, cDNA provides superior sensitivity compared to DNA; however, the barcode detection error is slightly higher (DNA: 13%, cDNA: 16%), possibly related to the increased frequency of errors being introduced by the reverse transcriptase during the cDNA synthesis [29].

Having confirmed limited skewing of the barcode representation at the RNA level at a transduction efficiency below 50%, we pursued in vivo validation of the labeling efficiency in nucleated and anucleated cells. We applied the Vav x Cre Rosa26 tdTomato mouse model, where all hematopoietic cells including platelets are tdTomato + [30]. In brief, FACS-purified lineage-Sca-1 + c-Kit + cells (LSK) from Vav x Cre Rosa26 tdTomato mice were lentivirally transduced, and after 24 h, we FACS-purified tdTomato + eGFP + cells and transplanted them into lethally irradiated recipients (Fig. 1D). We analyzed blood cell contribution of transplanted cells and observed chimerism level reaching 85–100% at week 16, which started to slightly decrease at week 20 in T cells. Importantly, we did not observe a loss of labeling in any particular lineage with the average silencing at 7.5% in all analyzed mature blood lineages (Fig. 1E–I), indicating the approach is appropriate for sensitive barcode detection in nucleated and anucleated LT-HSC progeny in long-term transplantation studies.

### Clonal composition of erythroid, myeloid cells, B cells, and platelets is stable over time

**Fig. 2:**
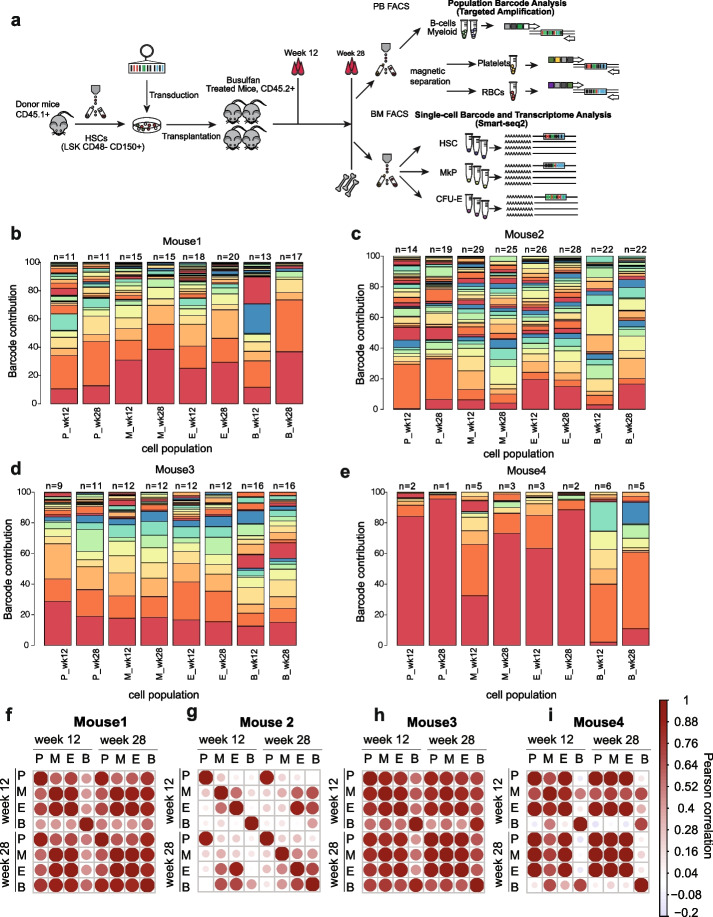
Clonal tracking in nucleate and anucleate blood cells. **A** Experimental design for in vivo multilineage RNA barcoding and single-cell (BM) and bulk (PB) readouts (for 4 recipient mice in 1 experiment). Prior BM harvest mice were injected with the carrier (PBS) 10 days before the termination. These animals constitute controls for the experiment in Fig. 4. **B**–**E** Stacked bar plots representing clonal composition in PB populations (P, platelets; M, myeloid cells; E, erythroid cells; B, B cells) at 12 and 28 weeks in 4 analyzed control animals, on top depicted is the Shannon count. Based on the cumulative relative abundance of ranked barcodes (saturation curves), we focused our analysis on the top 90% of barcodes. Colors have been reused between different mice depicted in different panels—the same color represents the same barcode within the same mouse; however, between different mice (**B**–**E**), the same color represents different barcodes. **F**–**I** Pearson correlation coefficient (generated using corrplot, R package) between platelet (P), myeloid cells (M), erythroid cells (E), and B cells (B) at 12 and 28 weeks post-transplantation. Exact *r* and *p* values are included in Additional file 5: Table S3

To study the clonal kinetics in platelets and nucleated cells in vivo, we transplanted on average 2500 barcoded LSK CD48 − CD150 + (LT-HSC) cells (Additional file 3: Fig. S2A) into 4 busulfan-treated recipients and measured the proportion of eGFP + cells in the population being analyzed (chimerism) (Fig. 2A). PB reconstitution was first assessed by measurement of eGFP + chimerism in platelets, RBCs, myeloid, and lymphoid cells at 12 and 20/28 weeks by FACS (Additional file 3: Fig. S2B-E). Prior to harvesting BM cells, mice were injected with the carrier (PBS) 10 days before the terminal analysis.

Subsequently, RNA from FACS-purified myeloid cells, B cells, and bead-purified RBCs and platelets at weeks 12 and 20/28 post-transplantation (Additional file 2: Table S1B) was used as input for barcode quantification by sequencing. Replicate samples from each lineage of the same mouse exhibited a high correlation of detected barcodes (mean *R* ^2^ = 0.95, Additional file 3: Fig. S2F-I), indicating robust barcode detection in the analyzed samples. This was especially important for platelet samples, since the mRNA content of these is 10,000 times lower than nucleated cells [31]. To perform the clonal analysis in PB, we ranked barcodes from most to least abundant in blood populations for each mouse and identified the “dominant” set of barcodes which collectively accounted for > 90% of total abundance. We focused our clonal kinetic analyses on these barcodes [28, 32] (Additional file 3: Fig. S2J-M).

We used Shannon counts to estimate the number of contributing clones in addition to a classic assessment of the clonal diversity within lineages estimated using the Shannon diversity index [28]. We have observed a wide range of clonal contributions in the dominant set ranging from 0.5 to 72% (Additional file 4: Table S2). We detected between 2–29 (week 12) and 1–28 (week 28) PB-dominant clones per mouse, with the number of clones dependent on the lineage analyzed (Fig. 2B–E). The lowest count being in platelets and the highest being in RBCs and B cells.

To measure the correlation between clonal composition in PB lineages at 12 and 28 weeks, we applied Pearson correlation coefficients between early and late time points. Overall, the correlations were high; however, we have observed that the barcode composition at week 28 between 4 analyzed PB lineages was higher compared to week 12 (Fig. 2F–I and Additional file 5: Table S3). This may relate to lower RNA content in myeloid cells and limited sample size at these time points. In mouse 4, all lineages at the 28-week time point have a high correlation. This is opposite to week 12 and likely relates to the expansion and clonal dominance of one clone, which was not as abundant at week 12, with the exception of B cells (Fig. 2I). Thus, our approach enables sensitive and stable detection of clonal output in all nucleated and anucleated PB lineages. At the same time, we show that tracking clones for longer time periods was essential for understanding the dynamics of clonal fluctuations.

### Multipotent clones are the most abundant and productive in the bone marrow during steady-state hematopoiesis

**Fig. 3:**
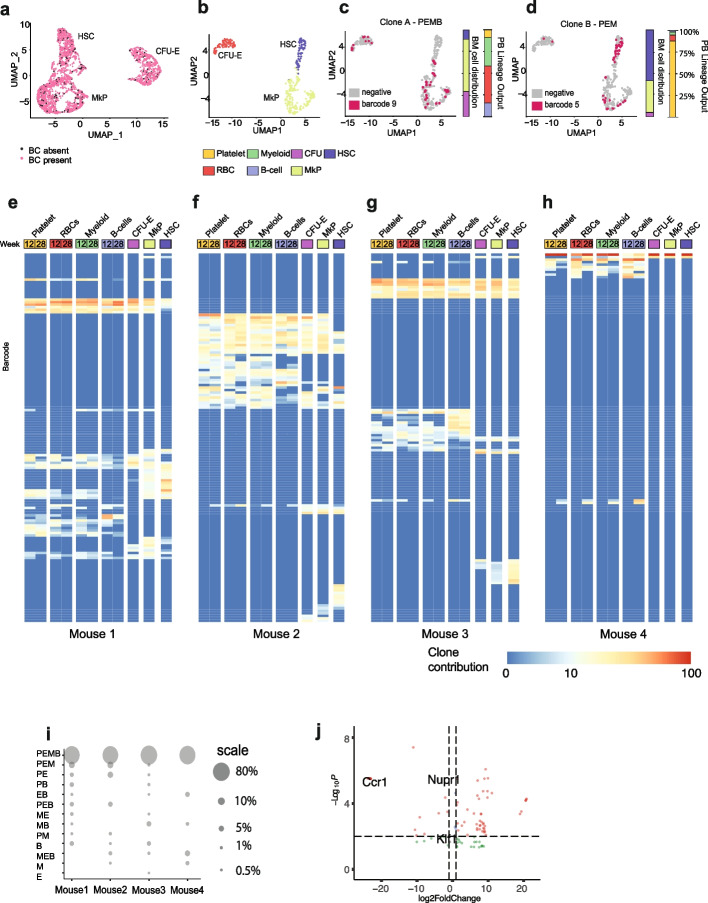
Clonal tracking in blood and bone marrow cells exerts high overlap between detected clones. **A** UMAP plot showing single-cell transcriptomes of sorted BM populations in which barcodes (BC) were detected (all cells which had over 50,000 reads, mitochondrial gene count < 10%, and barcode read count > 3 were included), analyzed cell numbers and cell QC (Additional file 5: Table S3, Additional file 8: Fig. S4B-E. **B** UMAP plot showing cell clusters based on single-cell transcriptomes of BM populations in mouse 2 (28 weeks post-transplantation). **C** UMAP plot representing clone A cells carrying barcode rank 9 in mouse 2 in the BM, bar plot representing PB lineage output (multilineage—PEMB, contribution to all 4 lineages > 0.089% at 2 consecutive time points) and the distribution of this barcode within the BM cell types profiled—depicted as a bar plot scaled from 0 to 100%, with labels at 25, 50, 75, and 100%. The adjacent bar shows the contribution of this clone to blood lineages, where total blood output is expressed as 100% and major ticks are 25, 50, and 75%. **D** As for **C**, but clone B is shown. This clone was classified as having platelet-erythroid-myeloid-restricted output (PEM, contribution to P, E, M > 0.089% and B lineages < 0.089% at 2 time points) and the distribution of this barcode within the BM cell types profiled—depicted as a bar scaled from 0 to 100%, with labels at 25, 50, and 75%. The adjacent bar shows the contribution of this clone to blood lineages, where total blood output is expressed as 100%, and major ticks are 25, 50, and 75%. **E**–**H** Clonal contribution of LT-HSC at 12 and 28 weeks post-transplantation in control animals injected with PBS. Heat maps representing the log fractional contributions of the top 90% most abundant contributing clones retrieved from each different bone marrow (BM) and peripheral blood (PB) cell lineage population, which were normalized per 1000. Each individual row represents the fractional contributions from an individual barcode (clone), and each individual column represents a sample. The rows are ordered by unsupervised hierarchical clustering using Euclidean distances to group barcoded clones together that manifest similar patterns of clonal contributions. The color scale on the right depicts the log10 fractional contribution size. Samples include Lin-Sca1 + cKit + CD150 + 48 − hematopoietic stem cells (LT-HSC), megakaryocytic progenitors (MkP), erythroid progenitors (CFU-E), platelets, erythroid cells (E), B cells (B), and CD11b + monocytes (Mac). **I** Bubble plot depicting the cumulative PB contribution of different types of clones in 4 control, vehicle-treated animals (mice 1–4). For the analysis, only dominant barcodes were included. To consider the clone as contributing to the lineage, it had to contribute > 0.089% at 2 time points. Abbreviations: PEMB, platelet-erythroid-myeloid-B cell; PEM, platelet-erythroid-myeloid; PE, platelet-erythroid; PB, platelet-B cell; EB, erythroid-B cell; PEB, platelet-erythroid-B cell; ME, myeloid-erythroid; MB, myeloid-B cell; PM, platelet-myeloid; B, B cell; MEB, myeloid-erythroid-B cell; M, myeloid; E, erythroid. **J** Volcano plot representing differentially expressed genes in LT-HSC clones producing only progenitors compared to LT-HSCs contributing to platelet and erythroid cells in PB, full list of differentially expressed genes Additional file 11: Table S6

Because of the strengths of our method to retrieve lineage information from anucleate cells, we focussed on their analysis and their nucleated progenitors (CFU-E and MkP) and LT-HSCs. To further investigate the relationships between the clonal composition in PB (Additional file 6: Fig. S3A-C) and the BM progenitor populations, we performed single-cell RNA-seq using Smart-seq2 [33] on eGFP + BM LT-HSCs and progenitors giving rise to anucleated lineages at week 28 post-transplantation (Additional file 3: Fig. S2A). This enabled comprehensive, full-length transcriptomic profiling of 2569 single cells from these phenotypically defined populations with the recovery of expressed cell barcodes in over 90% of cells (Fig. 3A–C, Additional file 7: Table S4). Each mouse had between 2 and 49 uniquely barcoded LT-HSC clones. This relatively low number of active clones may be related to the application of the busulfan as the BM ablation regimen [34] compared to total body irradiation used in other studies [17, 18, 35].

We subsequently mapped PB lineage output onto single FACS-purified and sequenced stem and progenitor cell transcriptomes (Fig. 3B–D, Additional file 7: Table S4, Additional file 8: Fig. S4B-E). This allowed the classification of individual LT-HSCs based on the composition of their lineage output (stable contribution > 0.089% to the lineage at two time points), as shown here (Fig. 3B, C) for two clones from mouse 2—clone A which demonstrated multilineage platelet/erythroid/myeloid/B cell (PEMB) output and clone B which showed more restricted platelet/erythroid/myeloid (PEM) output. The overlap between PB and BM revealed up to 70% of shared clones; however, each clone had different contributions to blood production and abundance in the BM (Fig. 3E–H). Out of all clones present in PB, multipotent clones contributed the most to mature blood lineages during steady-state hematopoiesis. These PEMB clones supported almost 80% of all PB output (Fig. 3I), in agreement with previous findings in nucleated cells [13, 35]. Conversely, platelet-biased clones were significantly less abundant in PB, and their contribution varied from 0.1 to 10% at a steady state following transplantation (Additional file 4: Table S2). The majority of clones that consist of only erythroid cells and B cells (EB) lack BM progenitors among sequenced cells. This is likely due to erythrocytes and B cells having a long half-life in PB. If during transplantation, only multipotent progenitors and not a true LT-HSC were transplanted the progenitor may have differentiated before the clonal analysis of the BM was performed (Additional file 9: Table S5A, B) and BM progenitors would therefore not be identified for these clones.

Due to the anucleate nature of erythroid cells and platelets precluding their direct clonal analysis using genomic DNA, the clonal composition of CFU-E and MkP progenitors has been widely used as a reliable proxy of the actual clonal composition in mature, circulating erythrocytes, and platelets [18, 36]. To evaluate the barcode overlap between CFU-E and blood erythroid cells or MkP and circulating platelets, we calculated the Jaccard distance between clones detected in mature cell type (erythrocytes or platelets) and progenitors (CFU-E or MkP). This analysis has revealed that 45.2% ± 12.1% of erythroid and 40.55% ± 15.7% of platelet clones would have been missed if the analysis would include only clones present in nucleated progenitor cell populations in BM (Fig. 3E–H, Additional file 10: Fig. S5A-D). There are multiple explanations for this. It may relate to the limited sampling of the in vivo progenitor cell populations in this study. It is also possible that some platelets may be generated from megakaryocytes that bypass the MkP stage [11, 12], or similarly, MkP/CFU-E progenitors may become exhausted, but leave progeny that is still detectable in PB. Alternatively, not all progenitors are supporting the generation of anucleate cells, in particular platelets, but have alternative functions, as already reported for megakaryocytes [23]. Therefore, we show that the most reliable clonal kinetics study of PB is a direct analysis of nucleated and anucleated cells present in the circulation.

Efficient barcode recovery from BM and PB lineages enabled parallel analysis of clonal composition in nucleated and anucleated cell types and their progenitor cells in individual mice (Fig. 3E–H). We observed functional LT-HSC heterogeneity in their reconstitution patterns. In mice 1–3, 40% of PB clones overlap with barcodes detected in progenitors, and the overlap is slightly lower with LT-HSCs (30%, Fig. 3E–H, Additional file 10: Fig. S5A–C). We also detected an extreme case of oligoclonality in mouse 4, where 70% of the PB and over 80% of the BM cells shared the same barcode (Figs. 2E and 3H).

Our approach allowed recovery of over 90% of clonal information from single BM cells, with an average of 1 M reads/cell. Analysis of barcode composition in PB and BM populations revealed up to 80% of shared clones between both tissues, which is significantly higher compared to previous barcoding studies using DNA [37]. The clonal composition among mice transplanted during the same experiment highlighted the high heterogeneity and repopulating potential of LT-HSCs. Importantly, our analysis has stressed the disparity between the BM and PB clonal composition in anucleated cells and their BM progenitors, highlighting the need for direct clonal assessment in these blood lineages, rather than using BM progenitors as a good approximation of clonal diversity in the blood.

We compared the transcriptomic phenotype of LT-HSC clones producing only progenitors (8.7% of all clones in Fig. 3E–H) to those actively contributing to circulating platelets and erythrocytes (18.7%, Fig. 3E–H), and detected 69 upregulated and 29 downregulated genes (Fig. 3J, Additional file 11: Table S6). Among these genes, we identified the transcription factors *Klf1* and *Nupr1*, and chemokine receptor *Ccr*1 as downregulated. Klf1 knock-out in fetal liver LT-HSCs has been shown to negatively regulate LT-HSCs and progenitor differentiation, in part through repression of the expression of IL-3, IL-5, and GM-CSF. It led to the accumulation of myeloid and erythroid progenitors but without the corresponding increased number of mature cells in circulation [38]. This suggests that the decreased expression of *Klf1* in LT-HSCs in our study could lead to the production of CFU-Es and MkPs that do not give rise to mature platelets or erythrocytes. We have observed this behavior in a group of LT-HSC clones producing MkP and CFU-E, while not contributing to platelets or erythroid cells in PB (Fig. 3E–H).

Growing evidence suggests *Nupr1* is a stress response transcription factor, which interacts with p53 to regulate the cell cycle and apoptosis and promote LT-HSC exit from quiescence [39]. Downregulation of this gene has been observed in LT-HSCs, actively supporting hematopoiesis in serial transplantation [18]. This suggests that decreased expression of *Nupr1* is a marker for LT-HSC clones actively supporting hematopoiesis upon transplantation; however, its downregulation may lead to decreased differentiation potential into mature PB lineages. Importantly, *Nupr1* is also upregulated in aged LT-HSCs and has been associated with their increased production of platelets and myeloid cells via regulating the expression of *Selp *[40]. *Ccr1* has been shown to enhance myelopoiesis and thus promote LT-HSC differentiation into mature myeloid cells upon infection [41]. It would suggest that decreased expression of *Ccr1* in LT-HSC and progenitor clones could inhibit their differentiation into mature, myeloid blood cell types (Fig. 3J, Additional file 11: Table S6).

### Platelet depletion forces repurposing of the lineage output in multipotent clones

**Fig. 4:**
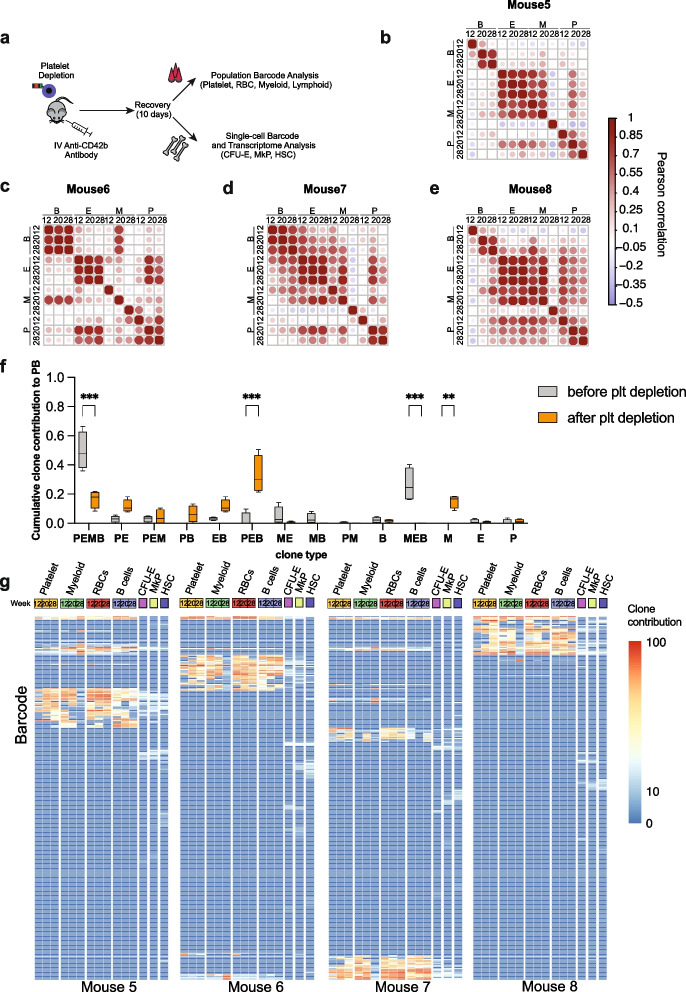
Acute platelet depletion causes dramatic changes in the clonal composition of the myeloid lineage causing the recruitment of completely new clones. **A** Experimental design for platelet depletion studies. **B**–**E** Pearson correlation coefficient between blood lineages (B, B cells; E, erythroid cells; M, myeloid cells; P, platelets) at 12 and 20 weeks (time points before the platelet depletion) and after platelet depletion (28 weeks). Exact *r* and *p* values (Additional file 5: Table S3). **F** Box plot representing clone contribution to PB before and 10 days after the platelet depletion. Statistical significance calculated using a 2-way ANOVA test with Bonferroni multiple comparison test (adjusted *p* values *** < 0.0001, ** < 0.001, * < 0.01, ns < 0.1). **G** Clonal contribution of LT-HSCs before and 10 days after platelet depletion. Heat maps representing the log fractional contributions of the top 90% most abundant contributing clones retrieved from each different bone marrow (BM) and peripheral blood (PB) cell lineage population, which were normalized per 1000. Each individual row represents the fractional contributions from an individual barcode (clone), and each individual column represents a sample. The rows are ordered by unsupervised hierarchical clustering using Euclidean distances to group barcoded clones together that manifest similar patterns of clonal contributions. The color scale on the right depicts the log fractional contribution size. Samples include Lin-Sca1 + cKit + CD150 + 48 − hematopoietic stem cells (LT-HSC), megakaryocytic progenitors (MkP), erythroid progenitors (CFU-E), platelets, erythroid cells (E), B cells (B), and CD11b + monocytes (Mac)

Next, we evaluated how the hematopoietic system would respond to acute, transient depletion of platelets. To assess cellular and multilineage clonal changes in response to acute platelet depletion, we induced acute thrombocytopenia by injecting anti-CD42b antibody into mice (Fig. 4A) transplanted with barcoded LT-HSCs 28 weeks post-transplantation (Additional file 12: Fig. S6 A-D) [42]. We confirmed the efficient platelet depletion 24 h post-treatment (Additional file 12: Fig. S6E). The kinetics of platelet replenishment has been extensively studied and requires 6–10 days for platelets to reach normal counts [10].

We collected BM and PB samples 10 days after treatment (28 weeks), at which point thrombocytopenia had ceased (Additional file 12: Fig. S6E) and determined PB and BM progenitor clonality at bulk and single-cell resolution, respectively (Fig. 4B–H). We compared the correlation among PB lineages before and after platelet depletion using the Pearson correlation coefficient, with two time points before (weeks 12 and 20) and one after the platelet depletion (week 28). We observed striking changes in the clonal composition of the myeloid lineages, rather than the platelet lineages, as we would have expected (Fig. 4B–E).

Interestingly, we did not notice decreased count or frequency of myeloid cells 10 days after the platelet depletion. Myeloid cells have a short half-life time in the peripheral blood (12.5 h to 7 days [43]), therefore requiring a constant input from upstream progenitor cells and LT-HSCs. This suggests that new clones have been recruited to solely produce myeloid cells. Indeed, when we looked into the clonal composition of the myeloid cells, the missing output from multipotent clones has been matched by a group of newly emerged clones exclusively producing almost 20% of myeloid cells in PB (Fig. 4F). Next, we compared the clonal composition in PB with clones detected in the BM and detected new LT-HSC clones activated to produce myeloid cells or platelets (Fig. 4F). This new subset of a platelet only producing clones contributed up to 5.8% of platelets (Fig. 4F). Besides, 57–81% of multipotent clones have been repurposed to produce platelets, erythroid, and B cells (Fig. 4F, G). To classify the clone as “repurposed,” it had to contribute over 0.089% (based on data obtained using unequally mixed cell line samples in vitro, Fig. 1C) into P, E, M, and B lineages at 2 time points before the platelet depletion, and after the perturbation, the clone contributed < 0.089% to myeloid cells (Fig. 4F). Repurposed clones were also found to have produced platelets, erythroid, and B cells (Fig. 4F, depicted as PEB (platelet-erythroid-B cell) clones). This likely relates to the half-life time of these two cell types: 22 days and 13–22 weeks for erythroid and B cells, respectively [44, 45]. The long half-life strongly suggests they were produced before the platelet depletion when the clones supported erythroid and B cell lineages (Fig. 4F). In total, by week 28, 50–65% (Fig. 4F) of all multipotent clones contributing to myeloid cells have stopped producing them and redirected their output to replenish platelets, reflected as the increased blood output of PEB clones from 5 to 40% after the intervention (Fig. 4F).

### Molecular signatures of repurposed multipotent and newly emerged myeloid clones in LT-HSC

**Fig. 5:**
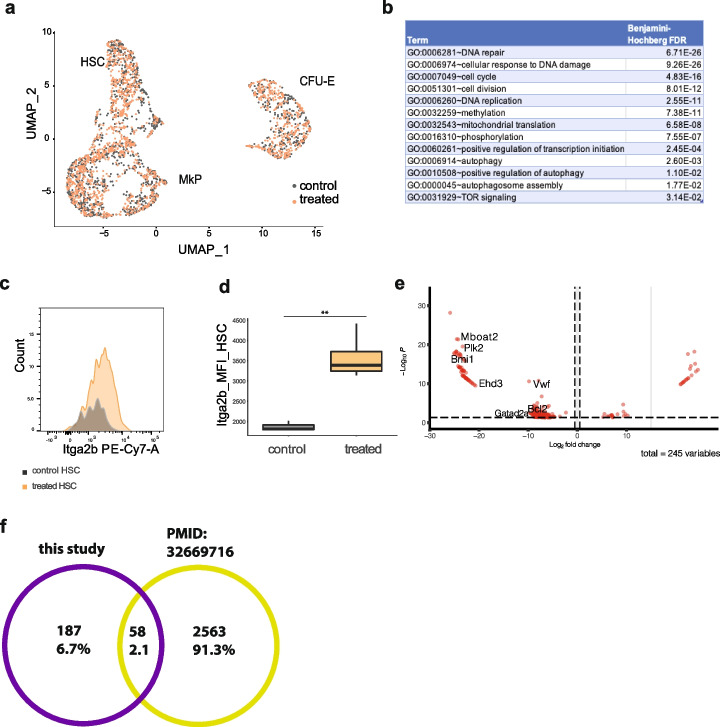
Depletion of platelets is also reflected in molecular signatures of LT-HSCs with different blood output. **A** UMAP plot representing the transcriptomic state of control and treated LT-HSC, MkP, and CFU-E (included cells with read count > 50,000, mitochondrial gene content < 10%); cell numbers in all 3 populations are included in Additional file 7: Table S4, and QC is included in Additional file 6: Fig. S3B-E. **B** Enriched processes in platelet-biased clones compared to control multipotent clones in platelet-depleted animals, shown are adjusted *p* values using the Benjamini–Hochberg multiple correction test (full list of processes Additional file 13: Table S7). **C** Itga2b expression in LSK CD150^+^48^−^eGFP^+^ cells measured 28 weeks + 10 days post-transplantation in vehicle-treated and platelet-depleted animals. **D** Increased mean fluorescence intensity (MFI) of Itga2b in LSK CD150 + 48-eGFP + in platelet depleted compared to vehicle-treated animals using Welch’s unequal variances *t*-test, *p* = 0.0073. **E** Volcano plot representing genes upregulated in newly emerged myeloid clones compared to PEB clones. **F** Gene overlap between platelet-depletion activated LT-HSC clones and clones activated in the serial transplantation in the study [18]. Differentially expressed genes were derived using the deseq2 R package. Pseudo-bulk samples were generated by adding gene expression matrices for cells belonging to active or low-output clones within one animal, which resulted in 4 samples (mice 5–8). These samples were used as input for deseq2

To investigate the striking clonal changes we observed upon platelet depletion further, we took advantage of the scRNA seq-data from the BM populations to link single-cell transcriptional profiles with PB output. Firstly, we performed unsupervised clustering on LT-HSC, MkP, and CFU-E cells from platelet-depleted and control (injected with a carrier) mice 10 days after perturbation (Fig. 5A). Cells from each type clustered together, regardless of the experimental group, indicating that the system has returned to steady state. Changes in PB clonality are not reflected by a global shift in stem cell and progenitor gene expression (Fig. 5A, Additional file 12: Fig. S6F). This suggests that there is either no or limited transcriptional memory of the challenge. It is plausible that short-term changes in the LT-HSC and MkP compartments caused by platelet depletion were missed in our dataset if they have occurred earlier than 10 days after the perturbation.

The overall transcriptional landscape has not changed, but there may be transcriptional changes in the reprogrammed multipotent (PEMB) clones affecting their functional output that is hidden when looking at the global picture. To do this, we performed differential gene expression analysis combined with the cells’ barcode information to select for clones whose functional output has switched after platelet depletion. From this analysis, we found that P/PE clones upregulated genes involved in DNA damage, DNA repair, DNA replication, cell cycle regulation, positive regulation of transcription, and DNA methylation (Fig. 5B, Additional file 13: Table S7). This suggests that LT-HSCs upon platelet depletion are exiting quiescence and become transcriptionally active. Similar mechanisms of metabolic rewiring are observed in LT-HSCs during inflammation, where high platelet consumption is also observed [46], and viral infection during emergency thrombopoiesis [11, 47]. During viral infection, a new phenotypic LT-HSC population has been described that had increased levels of ltga2b protein on the cell surface. We therefore reanalyzed our data and indeed observed increased protein expression of Itga2b in LT-HSCs from platelet-depleted animals when compared to carrier-treated controls (Fig. 5C, D). This suggests that LT-HSCs retain upregulated levels of Itga2b even when platelet counts are restored to control levels (Additional file 12: Fig. S6F). The changes here show that there are molecular changes occurring within specific clones.

Next, we asked what molecular changes are occurring between repurposed multipotent clones (PEMB before the platelet depletion and PEB after depletion) and newly appeared myeloid clones (M). We filtered the scRNA-seq data set and selected only LT-HSCs carrying barcodes identified in the peripheral blood as PEB or M clones. Differential gene expression analysis identified 245 (multiple test corrected *p* < 0.001, Additional file 14: Table S8) genes. Newly emerged myeloid clones have increased expression of genes encoding proteins involved in chromatin remodeling (*Bmi1*, *Gatad2a*, *Mboat2*), cell cycle regulation (*Plk2*), and apoptosis inhibition (*Bcl2*) compared to PEB clones (Fig. 5E, Additional file 14: Table S8). Myeloid clones also had high expression of *Vwf*, a marker for the platelet lineage which is expressed in platelet-biased LT-HSCs, MkP, mature megakaryocytes, and platelets [2, 10]. These cells may also start contributing to the platelet lineage at a later stage. *Mboat2* is an acetyltransferase involved in lipid metabolism, while *Gatad2l* is a member of the nucleosome remodeling and deacetylase complex (NuRD). This signature may indicate that these new myeloid clones are undergoing chromatin remodeling, which may change their lineage output.

We compared the gene signature of the LT-HSC clones we have identified that are activated upon platelet depletion with LT-HSC clones activated upon serial transplantation from a recent study [18]. The aim of this is to see if there are any common expression patterns or gene markers associated with the activation of LT-HSCs. Of the combined 2831 differentially expressed genes from the two studies, only 0.6% [16] genes were identified in both (Fig. 5F, Additional file 15: Table S9). This low overlap may result from the different biological cues leading to LT-HSC activation (platelet depletion versus total body irradiation and transplantation) utilizing different signaling pathways, total body irradiation causing changes to the LT-HSCs BM microenvironment [34, 48], and different cell capture, library construction, and analysis methods used.

**Fig. 6:**
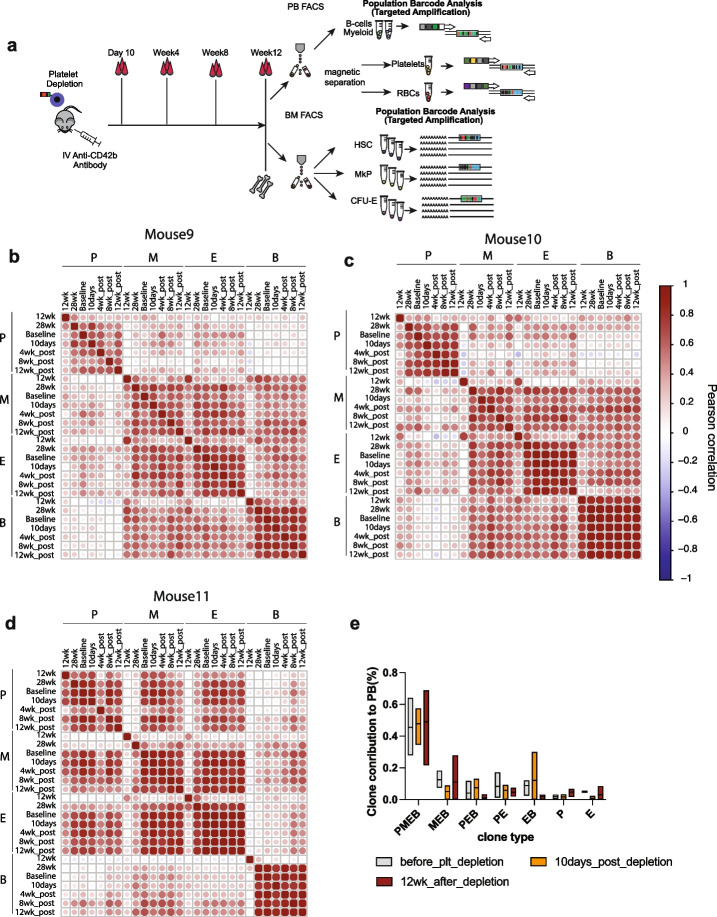
Platelet depletion leads to activation of myeloid output in P and PE clones. **A** Experimental model. Recipient mice treated with busulfan were transplanted with 2800 LT-HSCs, at a transduction efficiency of 33.4%, blood samples were collected at 12, 28, and 30 weeks post-transplantation (Additional file 16: Fig. S7 time point bas-baseline was taken 14 days before the platelet depletion at week 30), 10 days, and 4, 8, and 12 weeks post-platelet depletion along with bone marrow populations including GFP + LT-HSC, MkP, and CFU-E FACS—purified in bulk for clonal analysis. **B**–**D** Pearson correlation coefficient between blood lineages (B, B cells; E, erythroid cells; M, myeloid cells; P, platelets) at 12 and 28 weeks, baseline (30 weeks post-transplantation), time points before the platelet depletion, and after platelet depletion (10 days, 4, 8, and 12 weeks). Exact *r* and *p* values (Additional file 5: Table S3). **E** Box plot summarizing the contribution of clones to PB lineages (clone type abbreviations: PEMB-platelet-erythroid-myeloid-Bcell, PE-platelet-erythroid, MEB-myeloid-erythroid-B cell, EB-erythroid-B cell, P-platelet, E-erythroid, PEB-platelet-erythroid-B cell), line at mean

To analyze the long-term repopulating potential of clones activated upon platelet depletion, we have repeated the platelet depletion experiment for mice 30 weeks post-transplantation (labeled as baseline) and monitored the clonal kinetic changes in the blood up until 12 weeks post-intervention (Fig. 6A). In this experiment, we have observed less frequent clonal switching and activation indicated as higher Pearson correlation coefficients (Fig. 6B–E) compared to results from short-term analysis in Fig. 4F. We observed that between 13.5 and 18% of clones switched output or became activated upon platelet depletion. We noticed that data was more noisy compared to the short-term experiment (Fig. 4F). Still, we have observed similar trends: decreased PEMB and MEB clone contribution to PB at day 10 post depletion, which increased at a 12-week time point, this was correlated with increased output of PEB clones at 10 days and decrease at 12 weeks post-intervention (Fig. 6E). Again, 3 major trends (long-term emergence of new P and MEB clones, short-term emergence of PEB clones) could be observed, but in this instance, we did not observe statistical significance (Fig. 6E).

## Discussion

Detailed studies of HSC clonality in the hematopoietic system require reliable, stable, and heritable marking of LT-HSCs to trace them and their progeny through time. In this study, we have applied a novel RNA barcoding method to provide the quantitative and dynamic tracking of individual HSCs with regard to their lineage contribution and transcriptional signature during steady state and upon acute platelet depletion. In contrast to previous RNA barcoding studies of hematopoiesis [18, 19], our approach labels both nucleated and anucleate PB lineages including platelets. We have validated cDNA to be a more universal approach compared to previously published DNA barcoding methods [17] and a refined platelet collection method enabled their kinetic clonal studies. Importantly, we did not observe any significant silencing of the barcode in labeled PB cells. This suggests we were able to ensure robust, stable, long-term label retention in these cells, in turn enabling the quantitative study of the platelet lineage (Figs. 2F, G; 4B–E; and 6B–D).

Alongside our RNA barcoding method, we applied FACS purification and Smart-seq2 single-cell RNAseq [33] to obtain detailed information about rare LT-HSCs. The main advantage of using FACS purification followed by SmartSeq2 over droplet-based approaches is that it enables enrichment for rare cells and focussing on cells of interest before sequencing; the transcriptomes obtained using this approach are also more complex both in terms of the number of genes detected, but also in terms of sequence coverage, than those generated by droplet-based approaches. Finally, using index sorting, it is possible to combine the transcriptional information from SmartSeq2 with protein information obtained from FACS.

Our RNA barcoding method cannot easily be used for 3′ scRNAseq techniques combined as the barcode is located 1 kb upstream of the polyA signal [24]. However, it would be compatible with 5′ scRNAseq techniques. New droplet approaches including Vasa-seq could also resolve this issue [49]; however, none of these methods currently allows phenotypic selection of the cells of interest.

Our clonal analysis revealed that almost 80% of PB output at steady state comes from multipotent clones, which produce 50–80% of all platelets in the PB. Interestingly, we have detected less active LT-HSC clones contributing to hematopoiesis compared to previous studies (Fig. 2B–E) [13, 16, 17], which may be related to the different BM conditioning regimen: busulfan instead of total body irradiation. Busulfan is an alkylating conditioning agent that is given before transplantation and has been shown to allow high levels of donor chimerism [50, 51]. At the same time, busulfan retains cytokine milieu as suggested in a recently published study [34], in contrast to several published studies showing systemically increased production of inflammatory cytokines including IL-1, IL-6, and tumor necrosis factor (TNF), in the bone marrow microenvironment following irradiation. Importantly, these cytokine levels remain increased for at least 3 months [52–55] and further remodel the LT-HSC microenvironment thus influencing their output. In light of the emergence of the “cytokine storm” upon irradiation, it is possible that LT-HSC clones are exiting dormancy and actively contributing to hematopoiesis, while upon busulfan treatment, more LT-HSC clones stay dormant in the bone marrow. This is a plausible explanation for the decreased number of active clones detected in the PB in this study compared to previous work [14, 17, 35]. Montecino et al. [34] did not quantify the frequency of repopulating LT-HSCs (limiting dilution) upon busulfan conditioning of the BM; therefore, our study expands our knowledge on the number of clones in this transplantation setting.

We observed a high overlap between clones found in PB and BM; however, a fraction of clones in the PB were not detected in the BM. This may reflect the longevity of BM progenitor cell types supporting PB production at the time of our analysis or might result from limited BM sampling in our study (potentially lower abundance or asymmetrical distribution of these clones in the BM). Overall, we have observed a functional heterogeneity in PB output of LT-HSCs (Fig. 3E–H). In this experiment, cells were transplanted at high doses. LT-HSCs originating from the same pool exert a broad spectrum of repopulating patterns: highly polyclonal (Fig. 3E–G) or oligoclonal (Fig. 3H), where we observed a strong dominance of a single clone in 1 out of 4 animals transplanted in the same experiment.

In agreement with previous studies, multipotent clones were the major contributors to blood production [35] including platelets, while platelet-biased clones only produced 0.5–14.9% of all platelets at steady state. Platelet-biased LT-HSCs producing only a fraction of all platelets in our polyclonally repopulated mouse model are in contrast to single HSC transplantation studies [2]. Thus, it raises a possibility that platelet-specific LT-HSCs will only produce large quantities of platelets during the extreme stress of a single stem cell transplantation assay, while when transplanted with other LT-HSCs their contribution is limited.

Among other clones, we have observed a group of LT-HSCs which produced progenitors (MkP or CFU-E) but did not give rise to the mature blood cells. These progenitors constitute almost 50% of all clones detected in the progenitor compartment (Fig. 3E–H, Additional file 10: Fig. S5A-D). This emphasizes the importance of including mature PB lineage analysis, rather than just their BM precursors when studying the clonal kinetics in long-term studies of hematopoiesis. It also confirms that cell barcoding approaches capable of labeling all PB lineages are essential to observe the kinetics of stress responses in the hematopoietic system. Our molecular analysis and resulting gene signatures of LT-HSC clones producing platelets/erythroid cells compared to LT-HSC producing only progenitors (MkP and CFU-E) suggest that LT-HSC producing only progenitors have entered quiescence by downregulating expression of *Klf1* and *Nupr1*. Furthermore, the downregulation of *Ccr1* implies inhibition of the myeloid lineage differentiation program (Fig. 3J).

Acute platelet depletion revealed the remarkable plasticity and clonal dynamics of LT-HSCs in response to the clinically relevant stress of thrombocytopenia. From the literature, we would expect that platelet-biased LT-HSC clones would quickly and significantly increase their PB output [2] after perturbation and become the major contributors. However, here in polyclonally repopulated mice, our results show that it is easier for the system to repurpose the lineage output of 57–81% (depending on the animal) of already-present multipotent clones towards platelets. This repurposing was accompanied by the emergence of new myeloid clones at 10 days post-intervention (Fig. 4F). The need to replenish myeloid cells, but not B cells or erythroid cells, is most likely related to their similar short half-life time to platelets and the likely existence of a shared upstream progenitor cell, which is unable to sustain increased platelet production and replenish myeloid cells at the same time.

The discrepancy between single LT-HSC transplantation and our model in regard to platelet-biased LT-HSC contribution can in part be explained by the assay. In the single stem cell transplantation assay, the maximal repopulating potential of the LT-HSC is being evaluated, whereas in our polyclonally repopulated animals, there is a pool of LT-HSCs which interact with each other and coordinate their response upon stress. The latter scenario is closer to the actual situation in patients and therefore provides more physiological clues into LT-HSC plasticity and response to stress.

In the long-term follow-up study, although we have observed a limited number of new/switched clones, those still present at week 12 post-depletion were exclusively supporting platelets or started supporting other lineages (MEB clones) (Fig. 6E, Additional file 16: Fig. S7A).

## Conclusions

In summary, our novel barcoding approach enables true multi-lineage kinetic analysis of hematopoietic output in both nucleate and anucleate lineages including platelets. We have demonstrated its use in both steady state and following clinically relevant stress. Using scRNA-seq and barcode detection, effective in over 90% of single cells, the clonal output of individual cells can be linked with heterogeneity in gene expression and used to identify the molecular signatures of lineage biases. We demonstrate that acute platelet depletion enforces a switch in the clonal output from multipotent to exclusive platelet production and the activation of new, myeloid-biased clones. Using these results, we propose a model where multipotent clones (PEMB) support steady-state hematopoiesis; however, upon acute platelet depletion, they switch their output to platelets. In parallel, there is an activation of the myeloid differentiation program from low-output clones.

## Methods

### Mice

B6.SJL-Ptprca^Pep3b/BoyJ^ (CD45.1) mice were purchased from The Jackson Laboratory (Bar Harbor, ME). C57BL/6 J mice (CD45.2), B6.129S-Cybb^tm1Din/J^ mice were purchased from Charles River Laboratories. Animals were housed in a specific pathogen-free facility. All animal work in this study was carried out in accordance with regulations set by the United Kingdom Home Office and the Animal Scientific Procedures Act of 1986. Donor mice were 8 to 12 weeks old, recipient animals were 6–12 weeks old, and both genders were used for experiments. In experiments included in Figs. 2, 3, 4, 5, and 6, we have applied BM ablation by busulfan due to the available infrastructure at the University of East Anglia. This is an established method in our group [56–59].

### Cell isolation and preparation

BM isolation was prepared by isolating the tibia, femur, pelvis, spine, and sternum of each mouse. Bones were crushed in FACS media (phosphate buffer solution (PBS), Thermo Fisher, MA, USA) supplemented with 2 mM EDTA (Sigma Aldrich, Darmstadt, Germany) and 5% fetal bovine serum (Invitrogen, Darmstadt, Germany). Stem and progenitor cells were enriched from BM using CD117 (cKit) microbeads (Miltenyi Biotech, Bergisch Gladbach, Germany) prior to staining and sorting.

### Cell sorting

Antibody mixtures were prepared in 1 × FACS media and incubated with BM cells for 15–30 min at 4 °C. For flow cytometric cell sorting of BM cell populations, cells were resuspended in antibody mix, and cells were sorted directly into StemSpan (Stem Cell Technologies, Canada) with penicillin and streptomycin (GIBCO, MA, USA), hTPO, mSCF (Peprotech, MA, USA), or Smart-seq2 lysis buffer. Cell sorting was performed on a BD FACS Melody (BD Bioscience, CA, USA) or BD FACSAria Fusion (see Additional file 3: Fig. S2 for specific gating strategies, staining panel Additional file 17: Table S10A.

### Virus production

HEK293T cells (National Gene Vector Biorepository (NGVB), IN, USA) used for barcoding library virus product were cultured in DMEM (Thermo Fisher Scientific, MA, USA) with 10% FSC (Thermo Fisher Scientific), penicillin, and streptomycin (GIBCO, MA, USA) and incubated in 37 °C, in 5% CO_2_, with 95% humidity. Virus was prepared when the cells were within ten passages after they were obtained from NGVB, without further cell line authentication.

To generate the pEGZ2 lentiviral barcoding library [24], HEK293T cells (NGVB, IN, USA) were transfected with the pGIPZ-based library, pCMV-Δ8.9, and Vsv-g plasmids using Lipofectamine 2000 (Thermo Fisher, MA, USA) in Opti-MEM (Thermo Fisher, MA, USA). Twenty-four hours post-transfection, the medium was changed to DMEM (Thermo Fisher Scientific, MA, USA) with 10% FSC (GIBCO, MA, USA), penicillin, and streptomycin (GIBCO, MA, USA). Harvests were collected 48 and 72 h post-transfection and concentrated by centrifugation (8 h at 6000* g*, 4 °C). Cells were transduced with a barcoding library at an MOI of 50, defined as the titer on BaF3 [60] cells divided by the number of HSCs. This generated an HSC infection rate of ca. 45%.

### Transduction and transplantation

LT-HSC were sorted into StemSpan medium (StemCell Technologies, Vancouver, Canada) supplemented with 10 ng/ml mouse recombinant SCF, 100 ng/ml human TPO (both Peprotech, MA, USA), and penicillin/streptomycin (Thermo Fisher, MA, USA). Cells were spun down and resuspended in the viral supernatant [24] supplemented with the same concentrations of mSCF, hTPO, antibiotics, and polybrene (5ug/ml, Sigma Aldrich, Darmstadt, Germany). Cells were harvested 15–20 h later, extensively washed, counted, and resuspended in transplantation media (PBS + 5% FBS). Cells were immediately injected intravenously into mice (8–10 weeks old) preconditioned with busulfan 25 mg/kg/day for 3 days. The transduction efficiency was 47% (± 17%) in experiments. Due to the lack of infrastructure for total body irradiation at the University of East Anglia, we have applied an alternative method of BM ablation-busulfan, a method well established in our lab [56, 58, 59, 61, 62].

For evaluation of eGFP silencing LSK cells were FACS-sorted (BD FACS Aria Fusion, CA, USA) from VavCre x Rosa26tdTomato 8–10-week-old donors and transduced with barcoded viruses. Thirty hours after transduction, eGFP + viable cells were FACS-sorted (BD Fusion, CA, USA) and approximately 2500 eGFP + tdTomato + cells were transplanted into 8–12-week-old CD45.1 lethally irradiated (960 rad given as a split dose) recipient mice along with 200,000 CD45.1 + whole BM support cells. This initial experiment was performed at Karolinska Institutet, where the radiation source was readily available.

### Peripheral blood cell isolation

Mice were bled every 6–8 weeks. Blood was collected to EDTA coated tubes (Sarstedt, Numbrecht, Germany), gently shaken, and kept at room temperature. Next, tubes were spun down at 200* g* for 8 min at room temperature with no acceleration or brake. Platelet-rich plasma fraction was collected and used for platelet purification. The lower RBC fraction and the buffy coat were transferred to a new tube and mixed with 2% Dextran (Sigma Aldrich, Darmstadt, Germany) in 1:1 volumetric ratio with FACS media. A small fraction of the lower fraction was resuspended in isolation media (PBS + 0.1%BSA, 2 mM EDTA) and used for the purification of erythrocytes. Cells with Dextran were incubated for 15 min at 37 °C. Next, the upper phase was washed in FACS media and spun down (5 min, 500 g, 4 °C). The supernatant was aspirated and the cell pellet resuspended in ammonium chloride (Stem Cell Technologies, BC, Canada), incubated at room temperature, washed, and stained with antibody mix in Additional File 18: Table S11.

Cells were stained for 15–30 min at 4 °C, washed in FACS media, and sorted on BD FACS Melody (BD, CA, USA). Sorted myeloid cells were defined as Mac1 + (CD19-CD4/8-CD41-CD150-Mac1 + SSChi CD45-1 +) and CD19 + (Mac1-CD4/8-CD41-CD150-CD19 + CD45.1 +).

Platelet-rich plasma was resuspended in 100 μl of isolation media—PBS supplemented with 0.1%BSA, 2 mM EDTA, 0.001 M prostaglandin E2 (Millipore, Darmstadt, Germany) and 0.02 U/ml apyrase (Merck, Darmstadt, Germany) and stained with biotin-anti-mouse Ter119 antibody (clone-TER-119) and biotin-anti-mouse CD45 antibody (clone 30-F11, both from Thermo Fisher, MA, USA) for 15 min. Cells were washed in isolation media, spun down (5 min, 22 °C, 450 g) and resuspended in 100 μl of isolation media with Biotin Binder Dynabeads (Thermo Fisher, MA, USA). Cells were incubated for 15 min on the rotator at room temperature. Next, cells were placed on the magnet, and the supernatant was transferred to a new tube. An aliquot of the supernatant was subjected for the purity test by FACS using the following antibodies on BD FACS Melody, Additional file 18: Table S11.

Erythrocyte suspension was incubated with anti-mouse biotin CD41 (clone MWReg.30) and anti-mouse-biotin CD45 (clone 30-F11, both Thermo Fisher, MA, USA) for 15 min. Next, cells were washed in the FACS media, spun down, and resuspended in 100 μl of FACS media with Dynabeads Biotin Binder (washed in 1 ml of FACS buffer, resuspended in 100 µl before adding to the cell suspension, Thermo Fisher, MA, USA). Cells were incubated for 15 min on the rotator and placed on the magnet. The supernatant was transferred to the new tube, an aliquot of cells was used for the purity test using BD FACS Melody and stained with indicated antibodies (Additional file 18: Table S11).

### Platelet depletion

Mice transplanted with barcoded LT-HSC were intravenously injected with anti-CD42b antibody (R-300, Emfret, Mainz, Germany) at a dose of 2 μg/g body weight 28 weeks post-transplantation or a carrier (PBS, GIBCO). Blood samples were taken 24 h post-depletion to measure platelet count. A small blood sample was obtained by tail vein puncture with a 26-G needle, blood was collected in EDTA-treated tubes (Sarstedt, Numbrecht, Germany), stained with CD41-PC7 and run on FACS Melody with Sphero Counting Beads (Spherotech, IL, USA). Animals were sacrificed 10 days later, and the peripheral blood and bone marrow cells were subjected to the clonal analysis.

### Single-cell RNA sequencing library construction

Single cells from the bone marrow (LT-HSC, MkP, CFU-E) were FACS sorted (BD FACS Melody) into 2 μl Smart-seq2 lysis buffer [33]. Plates were stored at − 80 until further processing according to standard Smart-seq2 protocol [33]. Reverse transcription conditions: 42 °C for 90 min, 10 cycles 50 °C for 2 min, 42 °C for 2 min, hold at 4 °C; PCR amplification conditions: 98 °C for 3 min, 21 cycles: 98 °C for 20 s, 67 °C for 15 s, 72 °C for 6 min, final extension 72 °C for 5 min, hold at 4 °C. Amplified cDNA underwent bead clean-up in a 1:0.8 × volumetric ratio of Ampure Beads (Beckman Coulter, FL, USA) and eluted into 25 μl of elution buffer (EB, Qiagen, MD, USA). Selected libraries were analyzed on the Bioanalyzer using a High Sensitivity DNA chip (Perkin Elmer, MA, USA) to assess their quality. Next, cDNA was used for library construction using Nextera XT Kit (Illumina, CA, USA). The resulting libraries were pooled in batches of 384 and cleaned up using a 1:0.6 volumetric ratio of Ampure Beads (Beckman Coulter, FL, USA) and eluted into 25 μl of elution buffer (EB, Qiagen, MD, USA). Samples were barcoded during library preparation and 150-bp paired-end reads were generated on a NovaSeq6000 (Illumina, CA, USA).

### Blood sample processing for barcode recovery

Blood cell populations were sorted into FACS media, spun down, resuspended in RLT Plus Buffer (Qiagen, MD, USA), and stored at − 80 °C. Lysates were slowly thawed on ice and incubated with streptavidin-coated magnetic beads (DynaBeads, Thermo Fisher, MA, USA) as previously described [27]. The original G&T seq protocol has been modified—for barcoded transcript capturing on beads we have used a biotinylated barcode-specific primer (Rev6 5′-CGTCTGGAACAATCAACCTCTGG-3′). Obtained cDNA was used for the barcode amplification using forward primer containing a 9 nucleotide tag for multiplexing (Fwd3 5′-NNNNNNNNNCGGCATGGACGAGCTGTACAAG-3′) on the thermal cycler as follows: 95 °C for 3 min, then 30 cycles of 95 °C for 20 s, 63 °C for 15 s, 72 °C for 9 s, and finally 72 °C for 5 min. Amplified cDNA was cleaned up using a double-sided SPRI 1:1.2, collected the supernatant, transferred to a new tube, and added 1:2.5 × volumetric ratio of Ampure Beads (Beckman Coulter, FL, USA) and eluted into 25 μl of elution buffer (Buffer EB, Qiagen, MD, USA).

Libraries were used for PCR-free ligation of adapters (TruSeq, Illumina, CA, USA) following the manufacturer protocol with the modification for the bead clean up ratio used 1:2 × volumetric ratio (Ampure XT beads, Beckman Coulter, FL, USA). Obtained libraries were evaluated on Bioanalyzer and sequenced on NovaSeq6000 or a MiSeq (150 bp, paired-end, Illumina, CA, USA).

### Barcode recovery

#### Single cells

Obtained from the sequencer Fastq files were used for alignment to *Mus musculus* transcriptome version M38 using STAR aligner and count reads [63]. To obtain a single-cell expression matrix object, we used a custom R script. Subsequent analysis was performed in R using Seurat version 4.0.0 [64]. Cells showing gene counts lower than 50,000 reads and a mitochondrial gene expression percentage higher than 10% were excluded from further analysis. Within Seurat, data were normalized using the NormalizeData function (using the LogNormalize method and scale.factor = 10,000). The barcode sequence was recovered from scRNA-seq dat using BBduk which located the eGFP primer with Hamming distance = 1 (+ or − 1 nucleotide), subtracted this sequence and extracted the barcode structure. Barcode sequence and count were summarized in Additional file 3: Table S2. All barcodes with the read count below 3 were ignored. The probability of capturing 4 reads for the same barcode in 1 well purely by chance with the library consisting of 800 barcodes is (1/800)^4 = 2.44E − 12; thus, we considered it highly unlikely and applied in our pipeline.

To analyze differentially expressed genes we used R package deseq2 [65], using as input pseudo-bulk sample (generated as a sum of cells with a specific feature, for example, multipotent clones in mouse 1) preserving the biological variation, by keeping all mice as separate pseudo-bulk samples for this analysis.

#### Bulk PB sample barcode recovery

Barcodes for the PB samples were retrieved using custom scripts in python, as described previously [28]. We have detected 1 noninformative barcode (sequence: 5′-AAGGGCAACCTGGTAACCGATCTATGACACGATGTGTGACGGC-3′), which was excluded from all further analysis.

To obtain the Shannon count, we firstly calculated the Shannon index (H):where *s* is the total number of observed barcodes in the sample, *p* is the proportion of reads belonging to the *i*th barcode in the sample. Next, the Shannon index is converted into the Shannon count:

$$Sh = {e}^{H}$$

If all barcodes are equally distributed in the sample, the Shannon count is equal to the number of all barcodes detected in the sample. However, when the barcode distribution is skewed and their contribution is unequal, Shannon count is lower than the number of all barcodes, which makes Shannon count less sensitive to PCR noise [66]. To reliably detect barcodes, we have applied a cutoff of the top 90% of barcodes, since it has been shown that cellular barcoding is reliable at detecting medium-size and large clones; however, it struggles with the detection of small clones [66]. Thus, there have been several approaches to overcome this limitation. Some groups apply various tools depending on the structure of the barcode for semi-random barcodes such as Shannon count [28, 67, 68] or the top 90% of present barcodes identified in the sample [32], others apply the exact match to the constant fragment of the PCR handle downstream the barcode and recover a barcode sequence (by design random 17 nucleotides) allowing up to 4 mutations within the barcode [14, 16, 19, 69]. In this manuscript, we have applied the criteria of the top 90% of barcodes present in the sample. Barcode counts were retrieved using published pipeline [24] . Counts were normalized per 1000 and expressed as a fraction of total. The top 90% of barcodes contributing to PB were included in the analysis.

Relative measurement error has been calculated as:

$$\delta = \left| {\frac{{\upsilon_{A} - \upsilon_{E} }}{{\upsilon_{E} }}} \right| \cdot 100\%$$

where *δ* is the percent error, *υ*_A_ is the actual value observed, and *υ*_B_ is the expected value.

### Statistical analysis

Pearson correlation was applied as a measure of the distance between PB samples using the R package corrplot [70].

To calculate the barcode overlap between mature, anucleate cells, and their progenitors, we applied Jaccard distance:

$$J_{{{\text{dist}}}} = \frac{A\backslash B}{{A \cup B}}$$

where *A* is the barcode list in sample A, and *B* is the barcode list in sample B

Next, the distance was converted to a percentage and visualized using the Venn diagram. We have taken into consideration the list of barcodes (present/absent) disregarding the contribution of the clone.

Differences between the groups were calculated using the Student *t*-test for normally distributed variables or 2-way ANOVA using Prism (version 9.5.0). , , , .

## Supplementary Information

**Additional file 1: Fig. S1.** Correlation between barcode counts recovered from cDNA or gDNA. 1. Correlation between normalized read count recovered from cDNA and gDNA in single clones. Obtained raw reads in each sample were normalized per 1000 and plotted. 2. Assessment of cDNA and gDNA linear correlation using 3 barcoded clones depicted in panel A. The Pearson correlation coefficient is r=0.85. A pseudocount of 0.001 is used to plot non detected clones in gDNA samples. 3. Scatter plot showing the normalised barcode read count recovered from cDNAor gDNAfrom the same unequally mixed sample, included 2 technical replicates. 

**Additional file 2: Table S1.** A. 2-way ANOVA test with Tukey’s multiple comparisons test correction for barcode read count in unequally mixed samples retrieved from cDNA or gDNA (data shown in Fig. 1C). B. Cell numbers for nucleated and anucleated cell populations used for barcode retrieval. The purity for auncleate cells (% of CD45.1+cells and Ter119+ for P-platelets, %CD45.1+cells and CD150+CD41+ cells for E-red blood cells) was evaluated by FACS.

**Additional file 3: Fig. S2.** Stem cell FACS-purification strategy, contribution to blood lineages and barcode composition. A. Gating strategy to purify HSCfor transduction and transplantation experiments. Cells were transplanted into 4 busulfan treated recipients. B. - E. Contribution of transduced, eGFP+ LT-HSC in donor derived cells (except for platelets and erythroid cells) into 4 mature PB populations during  the course of the experiment, plots are showing the chimerism level in mice 1-4 (corresponds to clonal composition in mice depicted in Fig. 2 and 3). F.-I. Linear regression between 2 technical replicates for cDNA retrieved barcodes from 4 mature PB populations (P-platelets, E-erythroid cells, M-myeloid cells, B- B-cells) to assess the reproducibility of barcode recovery (displayed are normalised barcode read counts in 2 technical replicates in the same animal). Shown is the correlation between replicate samples in mice 1-4 (corresponds to Figure 2 and 3). J.-M. Contribution of clones (top 90% of all barcodes detected in PB) corrected for the chimerism level in analysed PB lineages (corresponds to animals in Fig. 2 and 3). We were able to detect clones contributing to platelet lineage at 0.1±0.11, erythroid 0.3±0.05, myeloid 0.2±0.1, B cell 0.2±0.12 (Fig. S1 J-M, Table S2).

**Additional file 4: Table S2.** Normalised barcode counts in PB and BM. Barcode counts were retrieved using published pipeline. Counts were normalised per 1000 and expressed as a fraction of total. Top 90% barcodes contributing to PB were included in the analysis.

**Additional file 5: Table S3.** Pearson correlation between barcode composition in peripheral blood lineages. Pearson correlation coefficient and p value for plots in Figure 2F-I, 4B-E, 6B-D calculated using corrplot r package.

**Additional file 6: Fig. S3.** Gating strategy for FACS purification of blood cell populations and purity of sorted cells. A. Gating strategy for PB cell population FACS-purification. Displayed are gates within the MNC/singlet/viable cell population. FACS definitions for purified populations: Mac1+, CD19+B. Purity of sorted Mac1+ cells evaluated by FACS C. Purity of CD19+ cells evaluated by FACS.

**Additional file 7: Table S4.** Numbers of eGFP+ LT-HSC, MkP and CFU-E cells recovered per mouse using Smart-seq2 protocol, which had over 50,000 reads/cell and less than 10% mitochondrial gene content, barcode read count > 3.

**Additional file 8: Fig. S4.** Gating strategy for FACS purification of transduced progenitors and stem cells, summary of single cell RNA sequencing including average read count, gene count and marker genes annotating cell clusters. A. Gating strategy for FACS-purification of single BM populations: LT-HSC, CFU-E and MkP, which were sorted as GFP+. B. Violin plots showing the distribution of read count per cell, number of genes per single cell and fraction of mitochondrial genes in single cells within 3 analysed BM populations C. UMAP plot representing computationally assigned clusters of cells based on their transcriptomes (Seurat clusters). D. UMAP plot representing 3 clusters of cells split by their FACS phenotype. E. UMAP plots representing the expression of marker genes for LT-HSC (Procr, Slamf1, CD48, Mpl, Selp), MkP (Mpl, Slamf1, Selp) and CFU-E (Hba-a1, Slc25a21).

**Additional file 9: Table S5.** Clone type overlap between mature anucleated cells and their bone marrow progenitors. A. Types of clones found in LT-HSC and PB, Progenitors and PBor in all 3 LT-HSC+Progenitors+PB, clone classification based on their output in PB, clone type name as in Fig.3I. B. Numbers of clones found in LT-HSC, Progenitor, LT-HSC and Progenitors, LT-HSC and PB, Progenitors and PBor in all 3 LT-HSC+Progenitors+PBto consider the clone as present in PB it had to contribute >0.089% to a lineage at 2 time points, otherwise clone was excluded from the analysis.

**Additional file 10: Fig. S5.** Barcode overlap between barcodes found in anucleate blood cells and their bone marrow progenitors. A.-D. Venn diagrams representing the overlap between dominant barcodes (top 90% barcodes identified in all PB samples at 12 and 28-weeks post transplantation) detected in anucleate cells (platelets or erythroid cells) and their BM progenitors (MkP or CFU-E) in mouse 1-4 calculated as Jaccard distance (details in Materials and Methods).

**Additional file 11: Table S6.** Differentially expressed genes between HSC producing only progenitors or progenitors and mature anucleate blood cells. List of DE genes (generated using deseq2 [56] between LT-HSC clones producing only progenitors or giving rise to progenitors and mature PB progeny.

**Additional file 12: Fig. S6.** Stem cell contribution to blood populations before and after the platelet depletion, heatmap representing hierarchical clustering of top 20 genes in vehicle or platelet depleted samples. A.-D. Chimerism level in mature PB populations in mouse 5-8. E. Platelet count 24-hours and 10 days after platelet depletion in mouse 5-8, carrier injected mouse served as a control (last 2 data points). F. Heat map representing an unsupervised, hierarchical cluster analysis of gene expression in  BM cells (read count >50 000, mitochondrial gene content<10%) for the top 20 genes enriched in each cluster, gene expression on a log2 scale from yellow to purple, cell populations are split by experimental group (vehicle treated/platelet depleted).

**Additional file 13: Table S7.** Differentially expressed genes between reprogrammed platelet-erythroid and multipotent clones. Full list of DE genes between reprogrammed platelet-erythroidand multipotent clonesupon platelet depletion using deseq2 and full list of enriched processes using DAVID bioinformatics [57].

**Additional file 14: Table S8.** Differentially expressed genes between reprogrammed platelet-erythroid-B cell clones and new myeloid clones. Full list of DE genes between reprogrammed platelet-erythroid-Bcelland newly emerged myeloid clonesupon platelet depletion using deseq2.

**Additional file 15: Table S9.** Differentially expressed genes between HSC activated by a platelet depletion or serial transplantation. DE genes between platelet depletion-activated LT-HSC clones and those activated in the serial transplantation from [18].

**Additional file 16: Fig. S7.** Clonal contribution of LT-HSC upon platelet depletion up to 12 weeks post intervention. A. Heat maps representing the log fractional contributions of the top 90% most abundant contributing clones retrieved from each different bone marrow (BM) and peripheral blood (PB) cell lineage population, which were normalised per 1000. Each individual row represents the fractional contributions from an individual barcode (clone), and each individual column represents a sample. The rows are ordered by unsupervised hierarchical clustering using Euclidean distances to group barcoded clones together that manifest similar patterns of clonal contributions. The color scale on the right depicts the log fractional contribution size. Samples include Lin-Sca1+cKit+CD150+48- hematopoietic stem cells (LT-HSC), megakaryocytic progenitors (MkP), erythroid progenitors (CFU-E), Platelets, Erythroid cells (E), B cells (B), CD11b+ monocytes (Mac). The “*” indicates platelet producing clones activated upon platelet depletion (contribution to platelet lineage <0.089% before the intervention or myeloid clones >0.089% activated to myeloid output upon intervention before the depletion these clones did not show a stable contribution to myeloid cells).

**Additional file 17: Table S10.** Staining panels for BM cell populations including antibody clone and conjugate.

**Additional file 18: Table S11.** Staining panels to FACS purify nucleate PB cells or evaluate the purity of anucleate cell populations.

**Additional file 19.** Review history.

## Data Availability

All data are available in the main text or the supplementary materials. The GEO submission number associated with the raw data presented in this manuscript is GSE188268 [71].

## References

[1] Osawa M, Hanada K, Hamada H, Nakauchi H (1996). Long-term lymphohematopoietic reconstitution by a single CD34-low/negative hematopoietic stem cell. Science.

[2] Carrelha J (2018). Hierarchically related lineage-restricted fates of multipotent haematopoietic stem cells. Nature.

[3] Gratwohl A (2010). Hematopoietic stem cell transplantation: a global perspective. JAMA.

[4] Weissman I (2005). Stem cell research: paths to cancer therapies and regenerative medicine. JAMA.

[5] Seggewiss R, Einsele H (2010). Immune reconstitution after allogeneic transplantation and expanding options for immunomodulation: an update. Blood.

[6] Pineault N, Boyer L (2011). Cellular-based therapies to prevent or reduce thrombocytopenia. Transfusion.

[7] Sieburg HB (2006). The hematopoietic stem compartment consists of a limited number of discrete stem cell subsets. Blood.

[8] Dykstra B (2007). Long-term propagation of distinct hematopoietic differentiation programs in vivo. Cell Stem Cell.

[9] Yamamoto R (2013). Clonal analysis unveils self-renewing lineage-restricted progenitors generated directly from hematopoietic stem cells. Cell.

[10] Nieswandt B, Bergmeier W, Rackebrandt K, Gessner JE, Zirngibl H (2000). Identification of critical antigen-specific mechanisms in the development of immune thrombocytopenic purpura in mice. Blood.

[11] Haas S (2015). Inflammation-induced emergency megakaryopoiesis driven by hematopoietic stem cell-like megakaryocyte progenitors. Cell Stem Cell.

[12] Morcos MNF (2022). Fate mapping of hematopoietic stem cells reveals two pathways of native thrombopoiesis. Nat Commun.

[13] Naik SH (2013). Diverse and heritable lineage imprinting of early haematopoietic progenitors. Nature.

[14] Wu C (2014). Clonal tracking of rhesus macaque hematopoiesis highlights a distinct lineage origin for natural killer cells. Cell Stem Cell.

[15] Gerrits A (2010). Cellular barcoding tool for clonal analysis in the hematopoietic system. Blood.

[16] Lu R, Neff NF, Quake SR, Weissman IL (2011). Tracking single hematopoietic stem cells in vivo using high-throughput sequencing in conjunction with viral genetic barcoding. Nat Biotechnol.

[17] Verovskaya E (2013). Heterogeneity of young and aged murine hematopoietic stem cells revealed by quantitative clonal analysis using cellular barcoding. Blood.

[18] Rodriguez-Fraticelli AE (2020). Single-cell lineage tracing unveils a role for TCF15 in haematopoiesis. Nature.

[19] Fan X (2020). Clonal tracking of erythropoiesis in rhesus macaques. Haematologica.

[20] Sender R, Fuchs S, Milo R (2016). Revised estimates for the number of human and bacteria cells in the body. PLoS Biol.

[21] Wright JH (1906). The origin and nature of the blood plates. Boston Med Surg J.

[22] Wright, JH. The histogenesis of the blood platelets. 1910;3(1).

[23] Sun S (2021). Single-cell analysis of ploidy and the transcriptome reveals functional and spatial divergency in murine megakaryopoiesis. Blood.

[24] Belderbos ME (2017). Clonal selection and asymmetric distribution of human leukemia in murine xenografts revealed by cellular barcoding. Blood.

[25] Shulman NR and Marder VJ. Similarities between known antiplatelet antibodies and the factor responsible for thrombocytopenia in idiopathic purpura. Physiologic, serologic and …. Ann NY Acad. Sci. 1965;124:499–542. https://nyaspubs.onlinelibrary.wiley.com/doi/abs/10.1111/j.1749-6632.1965.tb18984.x. 10.1111/j.1749-6632.1965.tb18984.x 5214832

[26] Gerlach C (2010). One naive T cell, multiple fates in CD8+ T cell differentiation. J Exp Med.

[27] Macaulay IC (2015). G&T-seq: parallel sequencing of single-cell genomes and transcriptomes. Nat Methods.

[28] Belderbos ME (2020). Donor-to-donor heterogeneity in the clonal dynamics of transplanted human cord blood stem cells in murine xenografts. Biol Blood Marrow Transplant.

[29] Arezi B, Hogrefe HH (2007). Escherichia coli DNA polymerase III ε subunit increases Moloney murine leukemia virus reverse transcriptase fidelity and accuracy of RT–PCR procedures. Anal Biochem.

[30] Georgiades P (2002). VavCre transgenic mice: a tool for mutagenesis in hematopoietic and endothelial lineages. Genesis.

[31] Angénieux C (2016). Time-dependent decay of mRNA and ribosomal RNA during platelet aging and its correlation with translation activity. PLoS one.

[32] Ferrari S (2020). Efficient gene editing of human long-term hematopoietic stem cells validated by clonal tracking. Nat Biotechnol.

[33] Picelli S (2014). Full-length RNA-seq from single cells using Smart-seq2. Nat Protoc.

[34] Montecino-Rodriguez E, Dorshkind K (2020). Use of busulfan to condition mice for bone marrow transplantation. STAR Protocols.

[35] Perié L, Duffy KR, Kok L, de Boer RJ, Schumacher TN (2015). The branching point in erythro-myeloid differentiation. Cell.

[36] Eisele AS (2022). Erythropoietin directly remodels the clonal composition of murine hematopoietic multipotent progenitor cells. Elife.

[37] Verovskaya E (2014). Asymmetry in skeletal distribution of mouse hematopoietic stem cell clones and their equilibration by mobilizing cytokines. J Exp Med.

[38] Hung C-H (2020). Negative regulation of the differentiation of Flk2− CD34− LSK hematopoietic stem cells by EKLF/KLF1. Int J Mol Sci.

[39] Wang T (2022). Loss of *Nupr1* promotes engraftment by tuning the quiescence threshold of hematopoietic stem cell repository via regulating p53-checkpoint pathway. Haematologica.

[40] Flohr Svendsen A (2021). A comprehensive transcriptome signature of murine hematopoietic stem cell aging. Blood.

[41] Broxmeyer HE, Cooper S, Hangoc G, Gao JL, Murphy PM (1999). Dominant myelopoietic effector functions mediated by chemokine receptor CCR1. J Exp Med.

[42] Bergmeier W, Rackebrandt K, Schröder W, Zirngibl H, Nieswandt B (2000). Structural and functional characterization of the mouse von Willebrand factor receptor GPIb-IX with novel monoclonal antibodies. Blood.

[43] Patel AA, Ginhoux F, Yona S (2021). Monocytes, macrophages, dendritic cells and neutrophils: an update on lifespan kinetics in health and disease. Immunology.

[44] Jones DD, Wilmore JR, Allman D (2015). Cellular dynamics of memory B cell populations: IgM+ and IgG+ memory B cells persist indefinitely as quiescent cells. J Immunol.

[45] Dholakia U, Bandyopadhyay S, Hod EA, Prestia KA (2015). Determination of RBC survival in C57BL/6 and C57BL/6-Tg (UBC–GFP) mice. Comp Med.

[46] Levi M, de Jonge E, van der Poll T, ten Cate H (1999). Disseminated intravascular coagulation. Thromb Haemost.

[47] Gekas C, Graf T (2013). CD41 expression marks myeloid-biased adult hematopoietic stem cells and increases with age. Blood.

[48] Curi MM, Cardoso CL, de Lima HG, Kowalski LP, Martins MD (2016). Histopathologic and histomorphometric analysis of irradiation injury in bone and the surrounding soft tissues of the jaws. J Oral Maxillofac Surg.

[49] Salmen F (2022). High-throughput total RNA sequencing in single cells using VASA-seq. Nat Biotechnol.

[50] Yeager AM, Shinn C, Pardoll DM (1991). Lymphoid reconstitution after transplantation of congenic hematopoietic cells in busulfan-treated mice. Blood.

[51] Hsieh MM (2007). Low-dose parenteral busulfan provides an extended window for the infusion of hematopoietic stem cells in murine hosts. Exp Hematol.

[52] Wilkinson FL (2013). Busulfan conditioning enhances engraftment of hematopoietic donor-derived cells in the brain compared with irradiation. Mol Ther.

[53] Xun CQ, Thompson JS, Jennings CD, Brown SA, Widmer MB (1994). Effect of total body irradiation, busulfan-cyclophosphamide, or cyclophosphamide conditioning on inflammatory cytokine release and development of acute and chronic graft-versus-host disease in H-2- incompatible transplanted SCID mice. Blood.

[54] Henry CJ (2015). Aging-associated inflammation promotes selection for adaptive oncogenic events in B cell progenitors. J Clin Invest.

[55] Youshani AS (2019). Non-myeloablative busulfan chimeric mouse models are less pro-inflammatory than head-shielded irradiation for studying immune cell interactions in brain tumours. J Neuroinflammation.

[56] Mistry JJ (2019). ROS-mediated PI3K activation drives mitochondrial transfer from stromal cells to hematopoietic stem cells in response to infection. Proc Natl Acad Sci U S A.

[57] Marlein CR (2019). CD38-driven mitochondrial trafficking promotes bioenergetic plasticity in multiple myeloma. Cancer Res.

[58] Moore JA et al. LC3-associated phagocytosis in bone marrow macrophages suppresses acute myeloid leukemia progression through STING activation. J Clin Investig. 2022;132 10.1172/jci153157 10.1172/JCI153157 PMC8884913 34990402

[59] Hellmich C (2022). p16INK4A dependent senescence in the bone marrow niche drives age-related metabolic changes of hematopoietic progenitors. Blood Adv.

[60] Palacios R, Henson G, Steinmetz M, McKearn JP (1984). Interleukin-3 supports growth of mouse pre-B-cell clones in vitro. Nature.

[61] Mistry JJ (2021). Free fatty-acid transport via CD36 drives β-oxidation-mediated hematopoietic stem cell response to infection. Nat Commun.

[62] Huang DW, Sherman BT, Lempicki RA (2009). Systematic and integrative analysis of large gene lists using DAVID bioinformatics resources. Nat Protoc.

[63] Dobin A (2013). STAR: ultrafast universal RNA-seq aligner. Bioinformatics.

[64] Papalexi E (2021). Characterizing the molecular regulation of inhibitory immune checkpoints with multimodal single-cell screens. Nat Genet.

[65] Love MI, Huber W, Anders S (2014). Moderated estimation of fold change and dispersion for RNA-seq data with DESeq2. Genome Biol.

[66] Bystrykh LV, Belderbos ME (2016). Clonal analysis of cells with cellular barcoding: when numbers and sizes matter. Methods Mol Biol.

[67] Belderbos M, de Haan G, Koster T, van der Velden V, Bystrykh L (2016). Subclonal evolution of pediatric acute lymphoblastic leukemia revealed by genetic barcoding. Exp Hematol.

[68] Jacobs S (2020). Quantitative distribution of patient-derived leukemia clones in murine xenografts revealed by cellular barcodes. Leukemia.

[69] Bramlett C (2020). Clonal tracking using embedded viral barcoding and high-throughput sequencing. Nat Protoc.

[70] Wei T, Simko V (2017). R package ‘corrplot’: visualization of a correlation matrix (Version 0.84).

[71] Wojtowicz EE, Mistry J, Uzun V, Hellmich C, Scoones A, Chin DW, Kettyle L, Grasso F, Lord AM, Wright DJ, Etherington G, Woll PS, Belderbos ME, Bowles KM, Nerlov C, Haerty W, Bystrykh LV, Jacobsen SEW, Rushworth SA, Macaulay IC. Panhematopoietic RNA barcoding enables kinetic measurements of nucleate and anucleate lineages and the activation of myeloid clones following acute platelet depletion. https://www.ncbi.nlm.nih.gov/geo/query/acc.cgi?acc=GSE188268. 10.1186/s13059-023-02976-z PMC10294477 37370129

