# Supplementary figures and images for: Panhematopoietic RNA barcoding enables kinetic measurements of nucleate and anucleate lineages and the activation of myeloid clones following acute platelet depletion
Source: Genome Biol. 2023 Jun 27;24:152. doi: 10.1186/s13059-023-02976-z (PMC10294477; doi:10.1186/s13059-023-02976-z)

Fig. S1

**a**

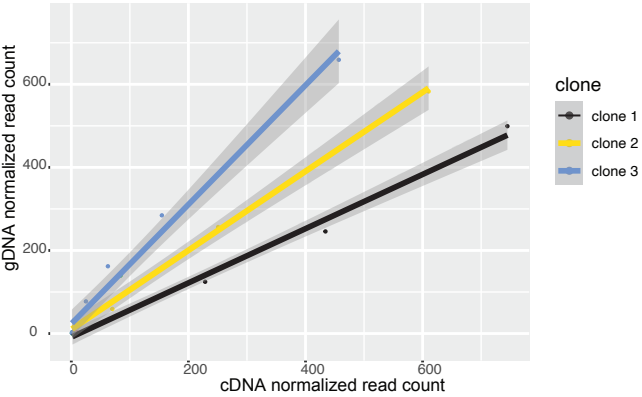

**b**

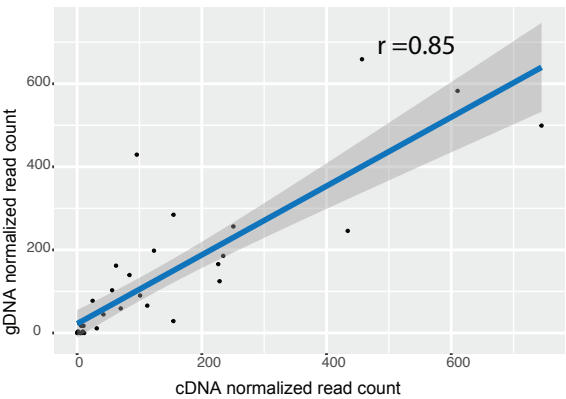

**c**

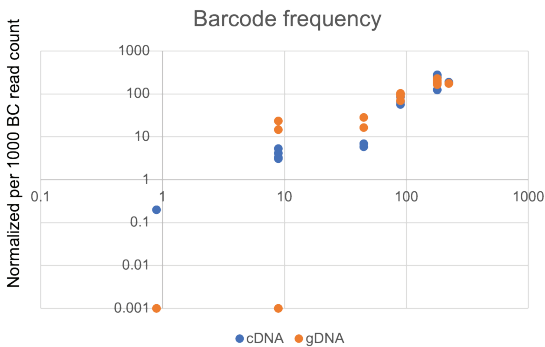

Supplement: Supplementary file 1 — Additional file 1: Fig. S1. Correlation between barcode counts recovered from cDNA or gDNA. 1. Correlation between normalized read count recovered from cDNA and gDNA in single clones. Obtained raw reads in each sample were normalized per 1000 and plotted. 2. Assessment of cDNA and gDNA linear correlation using 3 barcoded clones depicted in panel A. The Pearson correlation coefficient is r=0.85. A pseudocount of 0.001 is used to plot non detected clones in gDNA samples. 3. Scatter plot showing the normalised barcode read count recovered from cDNAor gDNAfrom the same unequally mixed sample, included 2 technical replicates. [file 13059_2023_2976_MOESM1_ESM.pdf]

Fig. S2

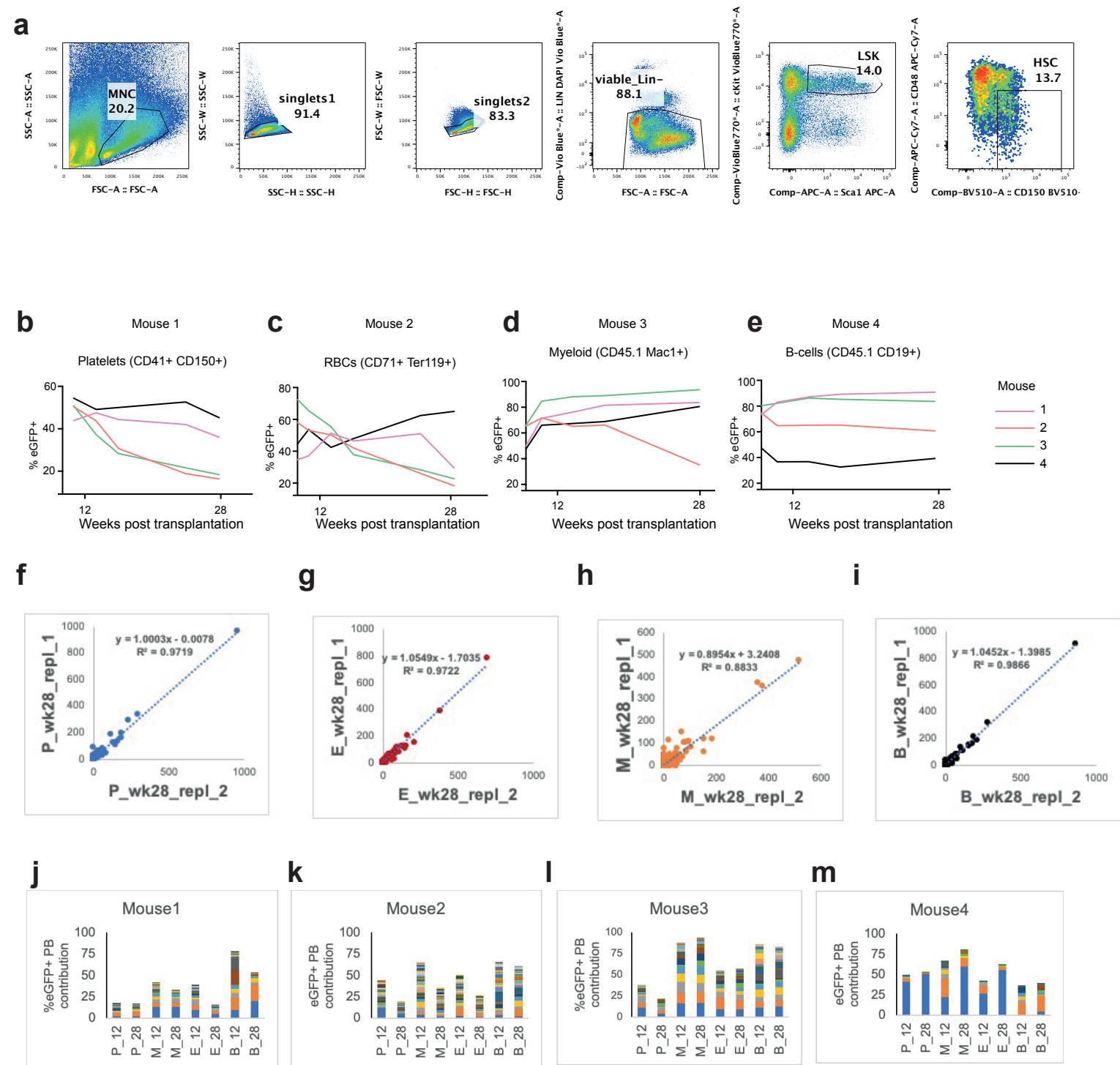

Supplement: Supplementary file 3 — Additional file 3: Fig. S2. Stem cell FACS-purification strategy, contribution to blood lineages and barcode composition. A. Gating strategy to purify HSCfor transduction and transplantation experiments. Cells were transplanted into 4 busulfan treated recipients. B. - E. Contribution of transduced, eGFP+ LT-HSC in donor derived cells (except for platelets and erythroid cells) into 4 mature PB populations during the course of the experiment, plots are showing the chimerism level in mice 1-4 (corresponds to clonal composition in mice depicted in Fig. 2 and 3). F.-I. Linear regression between 2 technical replicates for cDNA retrieved barcodes from 4 mature PB populations (P-platelets, E-erythroid cells, M-myeloid cells, B- B-cells) to assess the reproducibility of barcode recovery (displayed are normalised barcode read counts in 2 technical replicates in the same animal). Shown is the correlation between replicate samples in mice 1-4 (corresponds to Figure 2 and 3). J.-M. Contribution of clones (top 90% of all barcodes detected in PB) corrected for the chimerism level in analysed PB lineages (corresponds to animals in Fig. 2 and 3). We were able to detect clones contributing to platelet lineage at 0.1±0.11, erythroid 0.3±0.05, myeloid 0.2±0.1, B cell 0.2±0.12 (Fig. S1 J-M, Table S2). [file 13059_2023_2976_MOESM3_ESM.pdf]

Fig. S3

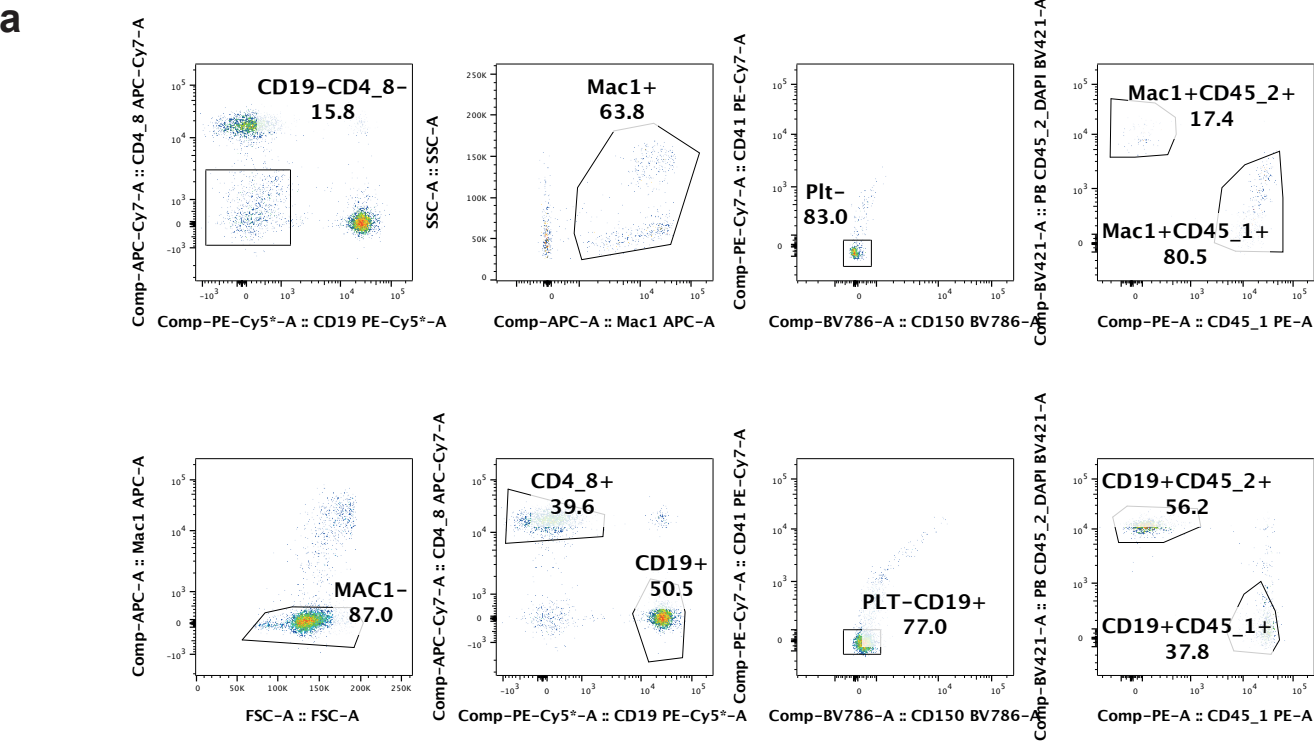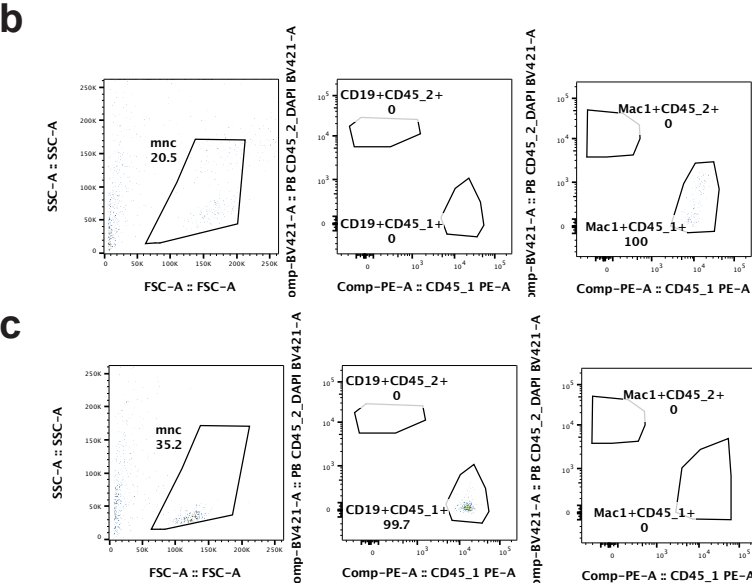

Supplement: Supplementary file 6 — Additional file 6: Fig. S3. Gating strategy for FACS purification of blood cell populations and purity of sorted cells. A. Gating strategy for PB cell population FACS-purification. Displayed are gates within the MNC/singlet/viable cell population. FACS definitions for purified populations: Mac1+, CD19+B. Purity of sorted Mac1+ cells evaluated by FACS C. Purity of CD19+ cells evaluated by FACS. [file 13059_2023_2976_MOESM6_ESM.pdf]

Fig. S4

**a**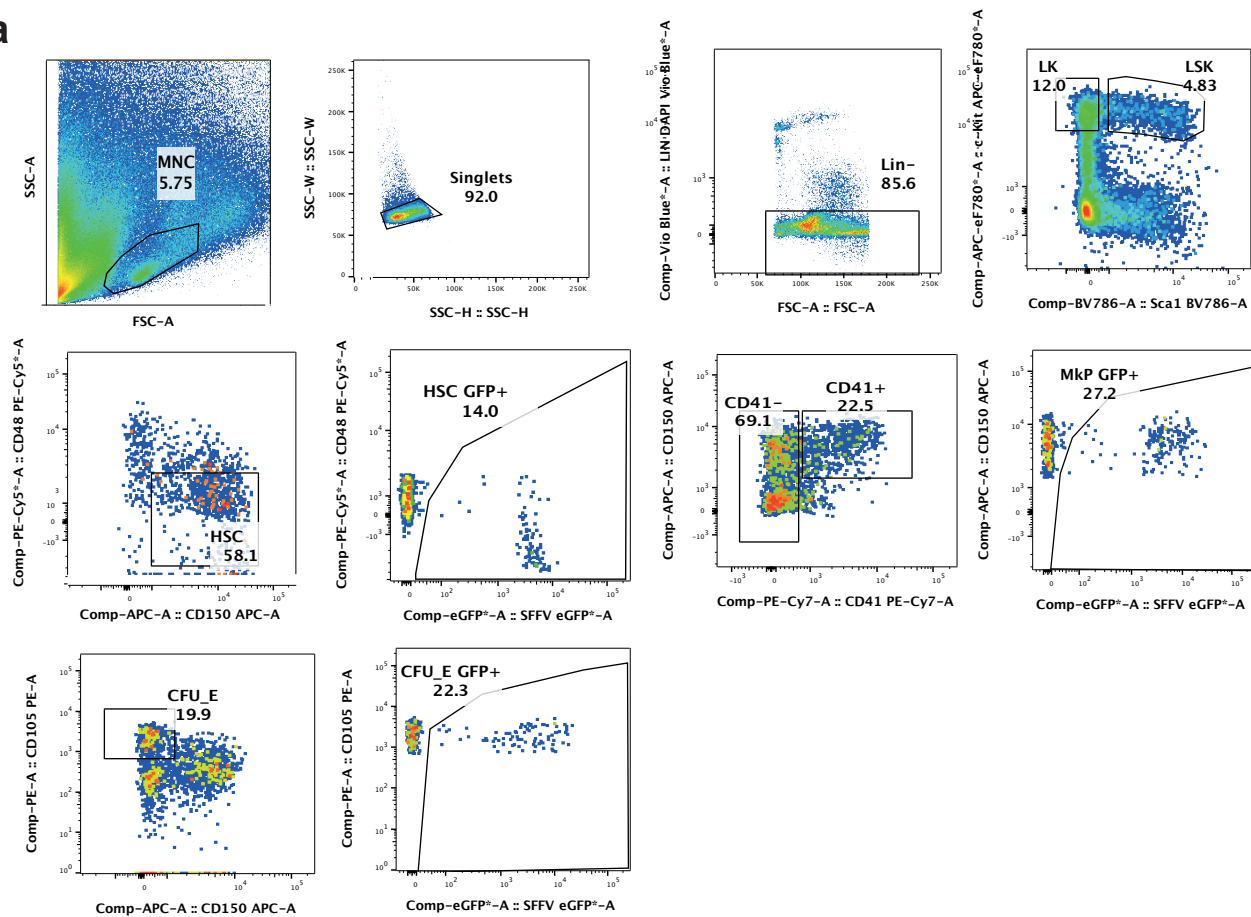**b**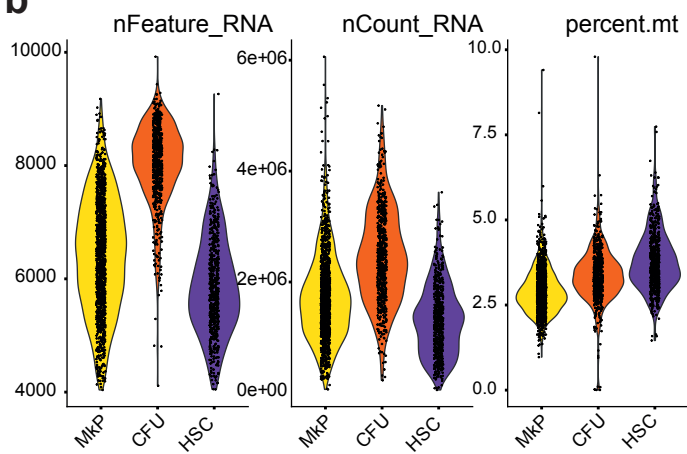**c**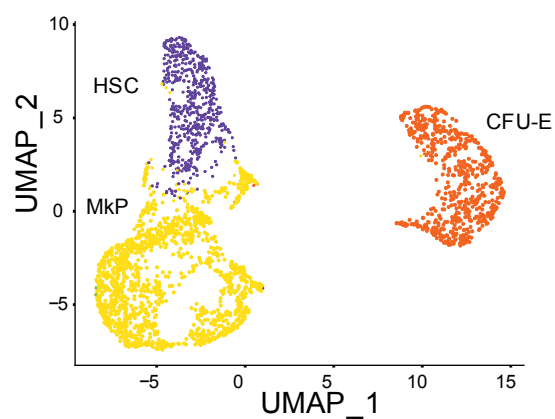**d**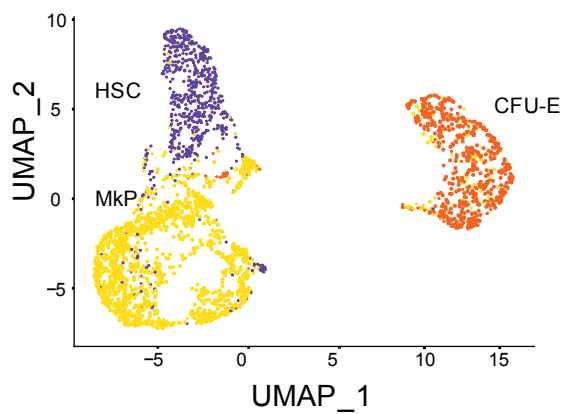**e**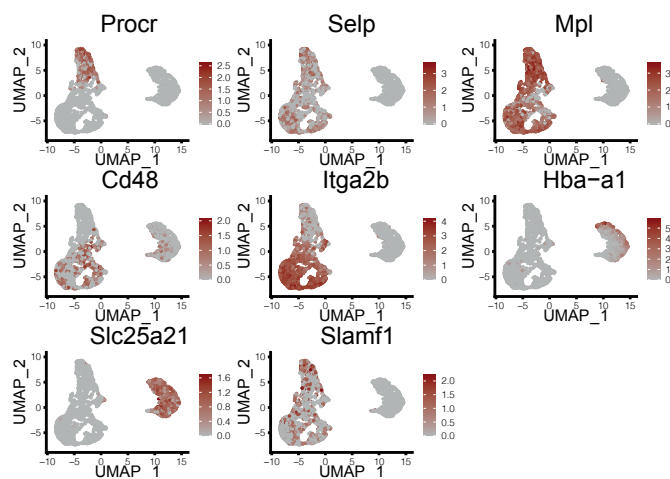

Supplement: Supplementary file 8 — Additional file 8: Fig. S4. Gating strategy for FACS purification of transduced progenitors and stem cells, summary of single cell RNA sequencing including average read count, gene count and marker genes annotating cell clusters. A. Gating strategy for FACS-purification of single BM populations: LT-HSC, CFU-E and MkP, which were sorted as GFP+. B. Violin plots showing the distribution of read count per cell, number of genes per single cell and fraction of mitochondrial genes in single cells within 3 analysed BM populations C. UMAP plot representing computationally assigned clusters of cells based on their transcriptomes (Seurat clusters). D. UMAP plot representing 3 clusters of cells split by their FACS phenotype. E. UMAP plots representing the expression of marker genes for LT-HSC (Procr, Slamf1, CD48, Mpl, Selp), MkP (Mpl, Slamf1, Selp) and CFU-E (Hba-a1, Slc25a21). [file 13059_2023_2976_MOESM8_ESM.pdf]

Fig. S5

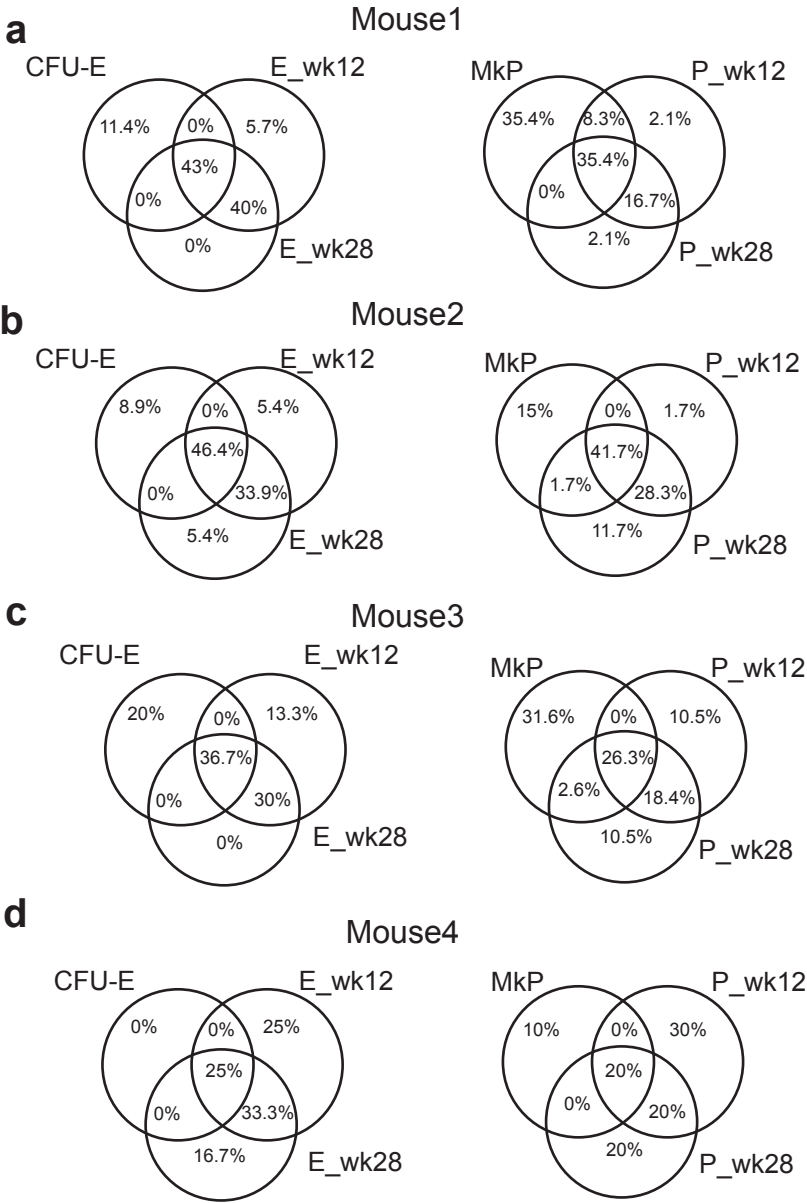

Supplement: Supplementary file 10 — Additional file 10: Fig. S5. Barcode overlap between barcodes found in anucleate blood cells and their bone marrow progenitors. A.-D. Venn diagrams representing the overlap between dominant barcodes (top 90% barcodes identified in all PB samples at 12 and 28-weeks post transplantation) detected in anucleate cells (platelets or erythroid cells) and their BM progenitors (MkP or CFU-E) in mouse 1-4 calculated as Jaccard distance (details in Materials and Methods). [file 13059_2023_2976_MOESM10_ESM.pdf]

Fig. S6

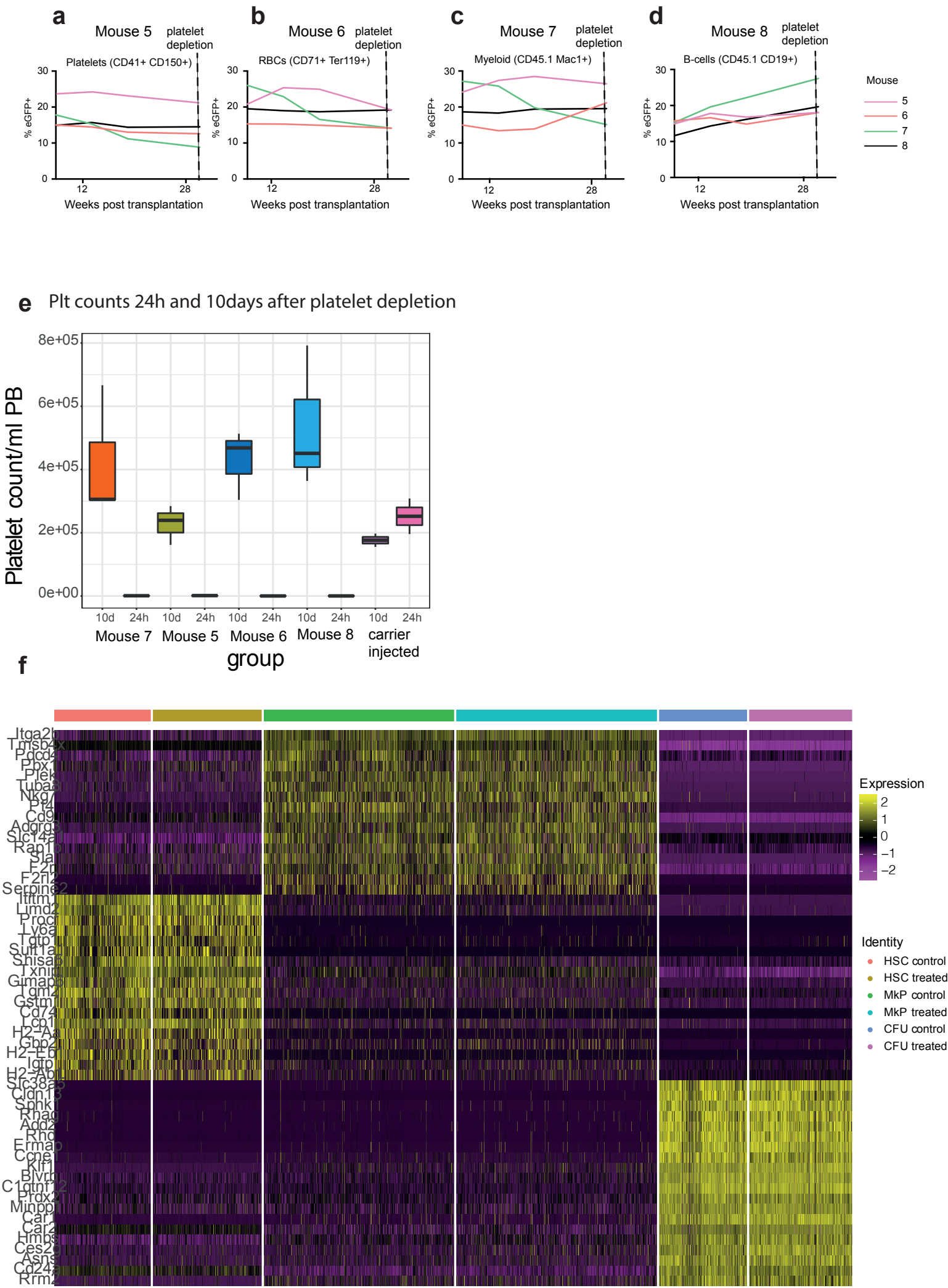

Supplement: Supplementary file 12 — Additional file 12: Fig. S6. Stem cell contribution to blood populations before and after the platelet depletion, heatmap representing hierarchical clustering of top 20 genes in vehicle or platelet depleted samples. A.-D. Chimerism level in mature PB populations in mouse 5-8. E. Platelet count 24-hours and 10 days after platelet depletion in mouse 5-8, carrier injected mouse served as a control (last 2 data points). F. Heat map representing an unsupervised, hierarchical cluster analysis of gene expression in BM cells (read count >50 000, mitochondrial gene content<10%) for the top 20 genes enriched in each cluster, gene expression on a log2 scale from yellow to purple, cell populations are split by experimental group (vehicle treated/platelet depleted). [file 13059_2023_2976_MOESM12_ESM.pdf]

Fig. S7

A

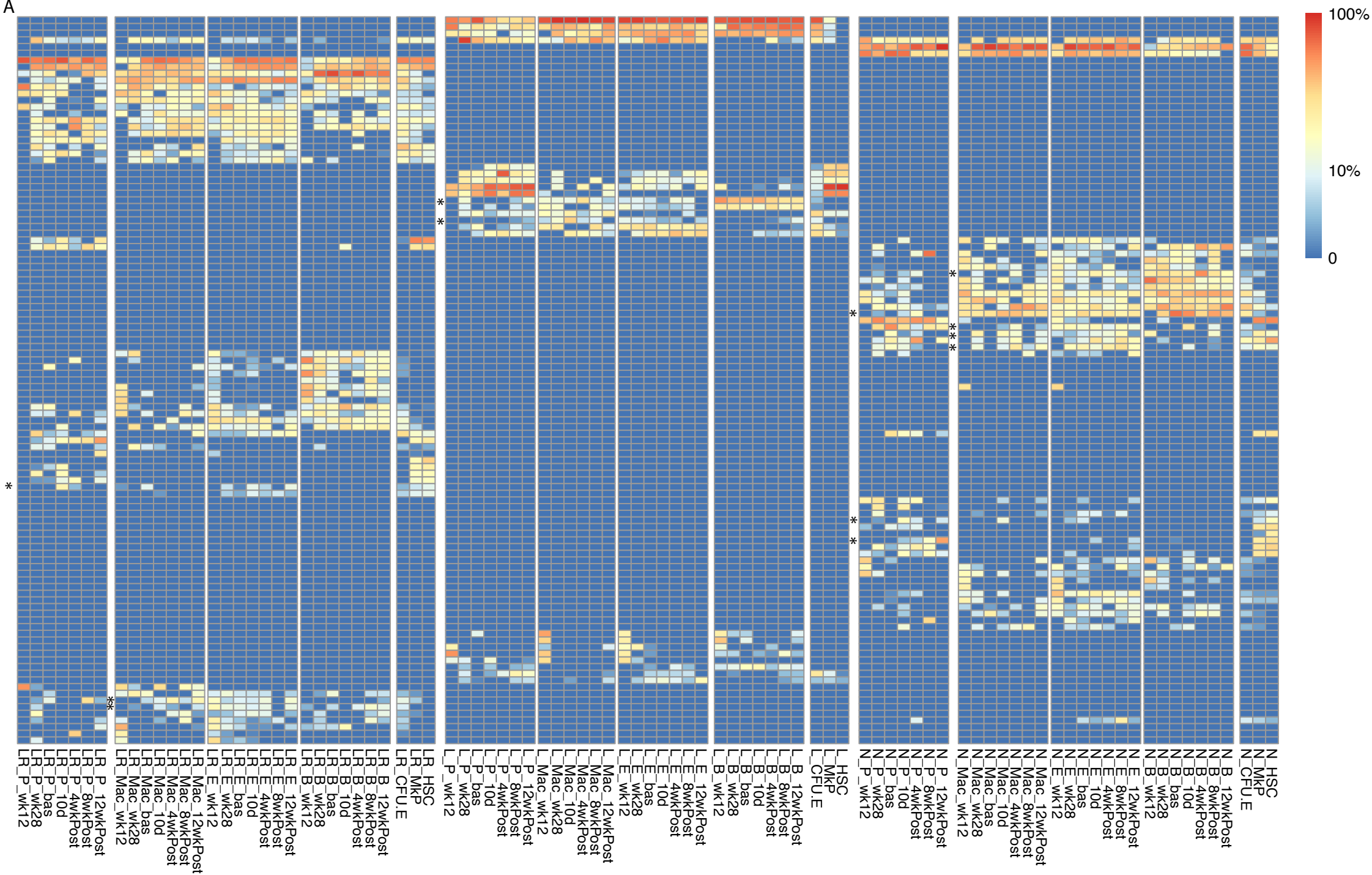

Supplement: Supplementary file 16 — Additional file 16: Fig. S7. Clonal contribution of LT-HSC upon platelet depletion up to 12 weeks post intervention. A. Heat maps representing the log fractional contributions of the top 90% most abundant contributing clones retrieved from each different bone marrow (BM) and peripheral blood (PB) cell lineage population, which were normalised per 1000. Each individual row represents the fractional contributions from an individual barcode (clone), and each individual column represents a sample. The rows are ordered by unsupervised hierarchical clustering using Euclidean distances to group barcoded clones together that manifest similar patterns of clonal contributions. The color scale on the right depicts the log fractional contribution size. Samples include Lin-Sca1+cKit+CD150+48- hematopoietic stem cells (LT-HSC), megakaryocytic progenitors (MkP), erythroid progenitors (CFU-E), Platelets, Erythroid cells (E), B cells (B), CD11b+ monocytes (Mac). The “*” indicates platelet producing clones activated upon platelet depletion (contribution to platelet lineage <0.089% before the intervention or myeloid clones >0.089% activated to myeloid output upon intervention before the depletion these clones did not show a stable contribution to myeloid cells). [file 13059_2023_2976_MOESM16_ESM.pdf]
