# Supplementary material for: Panhematopoietic RNA barcoding enables kinetic measurements of nucleate and anucleate lineages and the activation of myeloid clones following acute platelet depletion
Source: Genome Biol. 2023 Jun 27;24:152. doi: 10.1186/s13059-023-02976-z (PMC10294477; doi:10.1186/s13059-023-02976-z)
Supplement: Supplementary file 19 — Additional file 19. Review history. [file 13059_2023_2976_MOESM19_ESM.docx]

Review History

**First round of review**

**Reviewer 1**

**Were you able to assess all statistics in the manuscript, including the appropriateness of statistical tests used?** No

**Comments to author:**

In this manuscript by Wojtowicz et al., the authors perform analysis of mouse bone marrow regeneration using expressed lentiviral barcodes to track the behavior of individual hematopoietic stem cells (HSCs) during this process. In the manuscript, the authors have developed a method to accurately sample lentiviral barcode information from the RNA expressed in anucleated circulating cells, and they use this to understand the behavior of HSC clones after a lineage-specific injury (platelet depletion). In the end, the major claims of this article are that:

a) most HSCs steadily contribute to hematopoiesis;

b) barcode sampling from anucleated cells in the peripheral blood (Plt/RBCs) is superior to barcode sampling from bone marrow nucleated progenitors (MkPs/CFU-Es);

c) acute platelet depletion shifts multipotent HSCs to produce platelets exclusively, and causes the emergence of new myeloid-biased clones (from previously dormant or low-output clones).

I think that claim a) is not in and of itself novel as it is well known that HSCs contribute abundantly to hematopoiesis upon transplantation. At the same time, I believe that claim b) is slightly overstated, and perhaps misdirected: while I do agree that barcode sampling from anucleated cells is indeed superior, I think that it is not for the same reasons that the authors argue. Finally, I believe claim c to be the most novel and impactful part of this article, and quite sufficient to merit its publication. So much that I propose that it should be mentioned in the title itself, which, as it stands right now, does not really provide a clue as to why this study is novel and important.

The article is generally well written and mostly easy to follow, perhaps with some parts being bogged down with details that should be probably summarized or placed in the methods section (especially Figure 1 and 2).

The figure legends are extremely succinct and, combined with the plot legends and the little information in methods, it is hard to appreciate how to reproduce or interpret them. This makes for a confusing read of some of the figures (see my point-by-point comments below).

Overall, my conclusion is that this is a well-performed study that could be better presented and written to achieve the impact that such findings deserve. A few additional experiments are also suggested to even increase the potential impact of the paper's most novel findings.

I think that with a bit of revision (not a major overhaul) and a bit of sprucing up in the figures and legends, this manuscript would constitute an important contribution to the field.

My point by point follows.

1) It is not indicated why busulfan is the method of choice to analyze the clonal dynamics. What is the difference with lethal irradiation (as tested in Figure 1)? Why was it changed from Figure 2 on?

2) For 2500 HSCs transplanted, isn't the number of clones low? Do you think this is due to busulfan instead of irradiation? Also, there are way fewer Plt-biased clones, compared to multiple previous studies… could this also be due to the busulfan instead of irradiation?

3) I don't understand the barcode plots from figure 3 and 4. In the legend it says that the heatmap is showing the Spearman correlation between PB and BM… but then the scale is 0-10. A formula in the methods would make things clearer. What's being plotted is still unclear to me. Also, why are they called "control" animals in the legend? If these were processed together with those from the Plt-depletion and actually served as proper controls (injected with carrier, instead of anti-CD42d), then this should be made clear in the text, methods and legends.

4) The efficiency of barcode capture is excellent. Was Smartseq 2 required to increase coverage of the barcode? Is this "RNA" barcode sequencing amenable to 3'-biased methods (such as 10x genomics, inDrops, etc)?

5) Why were only LT-HSCs, MkP and CFU-E sampled by scRNAseq, instead of MPPs, GMPs, etc.? How many cells of each type were sampled? From how many mice?

6) In Supp Figure 3E-H, it's confusing what the Venn diagrams represent (and what the main text refers to). What does % mean? It seems to refer to the % of clones. But sampling is so completely different in PB vs scRNAseq BM, so wouldn't the % of cells (as estimated from the PB reads) be more accurate?

7) "Overall this data demonstrates the stochasticity in clonal composition of PB and BM cell populations in different animals transplanted in the same experiment." Could you better explain the meaning of this conclusion?

8) 1M reads per cell seems really challenging to achieve with non-Smartseq2 methods. Does the 90% barcode detection really depend on 1M reads per cell depth?

9) One thing that is interesting to check in the data is the existence of inactive and active HSC clones based on their output, similar to a recent previous report (ref. 17 in the manuscript). Just skimming the barcode data in Figure 3, it looks like some of these inactive HSCs are also seen but not followed up at all. It would be interesting to check whether the current study, using now busulfan instead of irradiation, reaches similar conclusions regarding active/inactive HSC clones and their signatures. Also, it seems interesting that when fewer clones engraft (mouse 4), this behaviour of active/inactive clones is no longer apparent, suggesting that only when a sufficiently diverse pool of HSCs engraft can these differences in HSC behavior arise. It would be interesting to compare the transcriptional signatures of HSCs activated by Plt depletion (Fig. 4-5 of present manuscript) with those from ref 17.

10) Instead of focusing so much on why PB is better than BM measurements on purely quantitative terms, I would encourage authors to focus on the unique and novel hypotheses that only their tool/approach can allow them to test. A unique aspect of proper sampling of anucleated cells that has never been done before is to compare clones by their capacity to sustain long-term production of these anucleated cells. Another uniquely addressable question is, for instance, why are some lineages so much more abundant in the BM than in the circulating Plt/RBC pool. In fact, are HSC clones that produce circulating Plt transcriptionally different from those that produce just BM MkPs/CFU-E but fewer circulating cells? There seems to be a few examples of both types of clones in the dataset shown (at least in mouse 2 and mouse 3). The other unique superiority of the technique is obviously shown in Figure 5, where dynamic shifts of clonal behaviors are observed through time within the same mouse, and then compared with transcriptomes of HSCs. Yet again, this dynamic capacity to sample all blood lineages from the PB within the same mouse (which I argue is the most important aspect of this technology, directly linked to the most novel and impactful results of this work, in figure 5) is somehow not the central argument given in the main text or the discussion.

11) "Cells from each type clustered together, regardless of experimental group, indicating that the changes in PB clonality is not reflected by a global shift in progenitor gene expression". I don't think this wording is correct. There could have been a shift in gene expression during the initial response (before the 10 days post-challenge), which was just not measured, because the researchers allowed the system to restore almost to the steady-state. What is actually being observed is that there is no global transcriptional memory of the challenge and thus the challenged and non-challenged cells are essentially indistinguishable by unsupervised clustering. The results from supervised clustering, equally, reveal that LT-HSCs that switched to platelet production during stress actually did retain a bit of a transcriptional memory of the challenge (even if globally the cell state is unaltered). This is actually one of the most interesting and novel aspects of this study, and it's really a pity that this wasn't also assayed after a longer period of time (say 30 days), which would better ensure that this is a proper transcriptional memory of the platelet-depletion.

12) I suspect that Figure 5f and 5g are mislabeled. Gray should be before plt depletion and orange should be after. This should be clarified in the legend as well. In fact, in general, the legends for figures 3-4-5 do not contain the necessary information to understand these plots.

13) Several figures and legends have other problems too and they might all benefit from a little sprucing up. For instance: some plot legends are too small to read; most scales and bars are not appropriately explained; the meaning of certain symbols in plots (such as ** in figure 5d) is not explained in legends and, if referred to statistical tests, there are not detailed in the legend either; some figure panels are not referenced in the main text (Fig 6b-c).

**Reviewer 2**

**Were you able to assess all statistics in the manuscript, including the appropriateness of statistical tests used? No**

**Comments to author:**

In this manuscript, the authors used RNA barcoding to study the blood production of individual hematopoietic stem cell clones after transplantation in mice. The authors claimed "for the first time including anucleate and nucleate lineages", but this is incorrect. A couple of published studies have already recovered barcodes from erythrocytes and platelets, including the one that was referenced (Reference #17). Most results presented in this manuscript validate known knowledge in the field. Probably the only new and interesting finding is that "acute platelet depletion shifts the output of multipotent HSCs to the exclusive production of platelets". However, the data presented did not directly and sufficiently show this point, nor the underlying mechanism. For example, what fraction of multipotent HSC clones become exclusively producing platelets? What are their differentiation and gene expression characteristics comparing to those multipotent HSC clones that do not exhibit this shift? There was only one data point analyzed after the platelet depletion. It would be interesting to see the dynamic changes in response to the platelet depletion in time course.

Major:

1. Figure 1b is not sufficient to demonstrate the correlation of barcode recovery from cDNA and from gDNA. It is important to compare individual barcodes, instead of the total read of all barcodes as in this figure.

2. "We reliably detected barcodes in as few as 1 cell (Figure 1) and clones that represent only 0.01% of the starting population were successfully detected, while they were missed at DNA level (Figure 1B)." (Page 3). These claims are not supported by the cited figures.

3. It is unclear why this study only considers barcodes that collectively account for >90% total abundance (Page 4).

4. "Fluctuations of clone size could be observed with some variation at week 12 and barcode composition becoming more similar among lineages at later time points (Fig. 1B, Suppl. Fig. 3A-D)." (Page 5) These claims are not supported by the cited figures.

5. Fig. 2F-I does not support the claim "These analyses demonstrate that clonal composition within cells of the same lineage is stable over time." (Page 5). For example, in Mouse 1, wk 12 M is closer to wk 28 E than to wk 28 M; wk 28 B is closer to wk 12 E than to wk 12 B; wk 28 E is closer to wk 12 M than to wk 12 E.

6. Figure 4G is missing but discussed in the result section: "This group of barcodes was also found in erythroid cells and B cells (Figure 4G, platelet-erythroid-Bcell clone, PEB clones), which can be explained by the half life time of the latter two cell types (22 days and 13-22 weeks respectively [31,32], strongly suggesting they have been produced before the platelet depletion, the time when the clone was multipotent and supported all analyzed blood lineages (Figure 4F, G)." (Page 8)

7. Figure 5b, it is unclear why the differential gene expression analysis was performed by comparing "multipotent clones in control animals with P/PE/PEB clones in platelet depleted animals". (Page 8). Have the authors compared (1) multipotent clones between control animals and platelet depleted animals; or (2) P/PE/PEB clones between control animals and platelet depleted animals?

8. Figure 5E, what is the definition of repurposed multipotent clones (PEB clones)?

9. Figure 5G is not discussed anywhere.

Minor:

1. "Relative measurement error of barcode detection at DNA level was 13%, while for cDNA was 16%." (Page 3). It is unclear how the "relative measurement error" is calculated.

2. Experimental detail is missing about the technical replicates in Suppl. Fig. 1f-i. Were the replicated samples processed separately starting from gDNA or cDNA isolation?

3. Figure 2b-e, does the same color represent the same barcode in each graph? This needs to be clarified in the figure legend.

4. Figure 3e-h and Figure 4f, what does the color show? What does the color key unit "scale" mean? How is "spearmen correlation" showed in these figures? These need to be clarified in the figure legend.

5. In figure 4b, wk28# E data dot is missing from the graph.

6. For scRNA seq analysis (Figure 5), were the cells harvested at week 28? How many cells of each type were harvested? How is the data quality?

7. Figure 5B, Log10 (FDR) color is difficult to read from the graph. Also, the full list of enriched GO terms needs to be provided in supplemental material.

**Authors Response**

**Point-by-point responses to the reviewers’ comments:**

**Reviewer #1:**

In this manuscript by Wojtowicz et al., the authors perform analysis of mouse bone marrow regeneration using expressed lentiviral barcodes to track the behavior of individual hematopoietic stem cells (HSCs) during this process. In the manuscript, the authors have developed a method to accurately sample lentiviral barcode information from the RNA expressed in anucleated circulating cells, and they use this to understand the behavior of HSC clones after a lineage-specific injury (platelet depletion). In the end, the major claims of this article are that:

a) most HSCs steadily contribute to hematopoiesis;

b) barcode sampling from anucleated cells in the peripheral blood (Plt/RBCs) is superior to barcode sampling from bone marrow nucleated progenitors (MkPs/CFU-Es);

c) acute platelet depletion shifts multipotent HSCs to produce platelets exclusively, and causes the emergence of new myeloid-biased clones (from previously dormant or low-output clones).

I think that claim a) is not in and of itself novel as it is well known that HSCs contribute abundantly to hematopoiesis upon transplantation. At the same time, I believe that claim b) is slightly overstated, and perhaps misdirected: while I do agree that barcode sampling from anucleated cells is indeed superior, I think that it is not for the same reasons that the authors argue. Finally, I believe claim c to be the most novel and impactful part of this article, and quite sufficient to merit its publication. So much that I propose that it should be mentioned in the title itself, which, as it stands right now, does not really provide a clue as to why this study is novel and important.

We thank the Reviewer1 for these points, we have changed the title of the revised manuscript to highlight novel findings enabled by RNA barcoding, which reads now : “**Panhematopoietic RNA barcoding enables kinetic measurements of nucleate and anucleate lineages and the activation of myeloid clones following acute platelet depletion”**

The article is generally well written and mostly easy to follow, perhaps with some parts being bogged down with details that should be probably summarized or placed in the methods section (especially Figure 1 and 2).

The figure legends are extremely succinct and, combined with the plot legends and the little information in methods, it is hard to appreciate how to reproduce or interpret them. This makes for a confusing read of some of the figures (see my point-by-point comments below).

Overall, my conclusion is that this is a well-performed study that could be better presented and written to achieve the impact that such findings deserve. A few additional experiments are also suggested to even increase the potential impact of the paper's most novel findings.

I think that with a bit of revision (not a major overhaul) and a bit of sprucing up in the figures and legends, this manuscript would constitute an important contribution to the field.

My point by point follows.

1) It is not indicated why busulfan is the method of choice to analyze the clonal dynamics. What is the difference with lethal irradiation (as tested in Figure 1)? Why was it changed from Figure 2 on?

We thank Reviewer1 for constructive comments, which we believe have strengthened our manuscript.

Due to the lack of irradiation source available at the University of East Anglia we had to find alternative ways to ablate bone marrow, therefore we have used busulfan. This method is well established in our lab [(1–4)](https://paperpile.com/c/6cJReo/1ljo4+6H9E1+iLzu5+RZ1NL) and has been also applied in this study, this information has been added to Methods section, **page 30** and the main text, **page 5**.

2) For 2500 HSCs transplanted, isn't the number of clones low? Do you think this is due to busulfan instead of irradiation? Also, there are way fewer Plt-biased clones, compared to multiple previous studies… could this also be due to the busulfan instead of irradiation?

Yes, it is possible that the bone marrow ablation by busulfan may have had an effect on the microenvironment, level of induced inflammation in the BM  and by this affected the number of active clones and the frequency of platelet-biased clones. This point was added to the main text**, page 5, Discussion page 12**,  Methods section page 32.

3) I don't understand the barcode plots from figure 3 and 4. In the legend it says that the heatmap is showing the Spearman correlation between PB and BM… but then the scale is 0-10. A formula in the methods would make things clearer. What's being plotted is still unclear to me.

We thank Reviewer1 for this comment. We apologise for the confusion and agree that the scale for the Spearman correlation was incorrect. As stated by the Reviewer the scale should have had values from -1 to 1 and therefore is counterintuitive to express the clonal contribution as -1. Thus to simplify the interpretation of heatmaps using our original input data we generated heat maps representing the log fractional contributions of the top 90% most abundant contributing clones retrieved from each different bone marrow (BM) and peripheral blood (PB) cell lineage population, which were normalised per 100. Each individual row represents the fractional contributions from an individual barcode (clone), and each individual column represents a sample. The rows are ordered by unsupervised hierarchical clustering using Euclidean distances to group barcoded clones together that manifest similar patterns of clonal contributions. This visualisation strategy has been recently applied in another barcoding study [(5)](https://paperpile.com/c/6cJReo/UvPqi). The colour scale on the right depicts the log fractional contribution size. Samples include Lin-Sca1+cKit+CD150+48- hematopoietic stem cells (LT-HSC), megakaryocytic progenitors (MkP), erythroid progenitors (CFU-E), Platelets, Erythroid cells (E), B cells (B), CD11b+ monocytes (Mac). This explanation has been added to the legends, Figure 3E-H and 4H.

Also, why are they called "control" animals in the legend? If these were processed together with those from the Plt-depletion and actually served as proper controls (injected with carrier, instead of anti-CD42d), then this should be made clear in the text, methods and legends.

Control mice were injected with a carrier (PBS) and it has been added to main text, page 5, Figure legend 2 and Methods section, page 25.

4) The efficiency of barcode capture is excellent. Was Smartseq 2 required to increase coverage of the barcode? Is this "RNA" barcode sequencing amenable to 3'-biased methods (such as 10x genomics, inDrops, etc)?

SmartSeq2 (SS2) was required for the single cell transcriptome capture and analysis of differentially expressed genes. Simultaneously during SS2 we have also captured the lineage information, since eGFP transcript is polyadenylated and amenable for standard SS2 protocol without requirement of a barcode specific capture probe.

SS2 is a more efficient approach for rare cells, since they can be efficiently enriched applying  FACS and directly deposited into plates compared to DropSeq-based protocols. Furthermore,  SS2 is better suited to obtain more detailed information about rare LT-HSC and it preserves the FACS phenotype of analyzed cells, therefore we solely  do not rely on transcriptomic classification of these cells.

 To the best of our knowledge our method is amenable to 10X 5’ 10X kit as recently published [(6)](https://paperpile.com/c/6cJReo/cGv2b). Alternatively a custom primer for barcode specific capture can be added together with standard oligo-dT in 3’ DropSeq. Another possibility to simultaneously capture the lineage and transcriptome information is application of Vasa seq[(7)](https://paperpile.com/c/6cJReo/MbNg). We did not include this validation experiment, since it is beyond the scope of the current manuscript.

5) Why were only LT-HSCs, MkP and CFU-E sampled by scRNAseq, instead of MPPs, GMPs, etc.? How many cells of each type were sampled? From how many mice?

Having in mind the strengths of our method for reading the lineage information in anucleated cells we have prioritised  MkPs, CFU-E progenitors and LT-HSC-which we sorted in triplicate (to have spare plates in case of any issues, we may have encountered during the cDNA synthesis or library construction). After sorting single cells into these plates, unfortunately, it was not possible to afford an additional sort of other progenitor populations eg.GMP or MPP. Due to the sorter set up when sorting in single cell mode it is possible to purify only 1 population at the time, all other cells are not recoverable.

With 10X all of these cell populations can be included.

Information on the exact cell number form each population analyzed per animal is included in Suppl. Table 3, the quality of assayed cells is included in Suppl. Figure 4B-E.

6) In Supp Figure 3E-H, it's confusing what the Venn diagrams represent (and what the main text refers to). What does % mean? It seems to refer to the % of clones.

In these panels we have presented the barcode overlap between Platelets at 2 time points and MkP or erythroid cells and CFU-E.

Barcode overlap between samples was calculated using Jaccard distance:

where

A-is the barcode list in sample A

B-barcode list in sample B

Next, the Jaccard distance was converted to percentage and visualized using the Venn diagram. We have taken into consideration the list of barcodes (present/absent) disregarding the contribution of the clone. This information has been included in the Methods section (Methods, page 36).

But sampling is so completely different in PB vs scRNAseq BM, so wouldn't the % of cells (as estimated from the PB reads) be more accurate?

Comparing absolute counts in the bone marrow and population counts (Shannon count) in the peripheral blood may seem to be problematic at first, however both ways of counting provide overlapping lists of barcodes and have the same limitation of detecting small clones.

We want to note, that the conversion between the clone size in the bone marrow and blood is more complex and to perform it one would need to consider turn over rate of the progenitor subsets, bone marrow anatomical locations included in the analysis-due to asymmetrical location of clones across the skeleton, differentiation potential and net production of mature cells by each of these clones. For these reasons we have performed a direct comparison between PB and BM counts.

7) "Overall this data demonstrates the stochasticity in clonal composition of PB and BM cell populations in different animals transplanted in the same experiment." Could you better explain the meaning of this conclusion?

We wanted to emphasize the functional heterogeneity of LT-HSC within the same experiment. Provided example presents 2 different scenarios, where mice transplanted with approximately the same dose of barcoded cells can have completely different repopulation pattern-highly polyclonal (mice 1-3) or oligoclonal (mouse 4), where we observed a strong dominance of a single clone. We have rephrased this sentence and included it, page 8.

8) 1M reads per cell seems really challenging to achieve with non-Smartseq2 methods. Does the 90% barcode detection really depend on 1M reads per cell depth?

To our understanding non-SmartSeq2 methods are compatible with our approach, as stated in  point 4.

Using our dataset we have tested the correlation between the barcode read count and the total read count per cell -it is R2=0.71, which may suggest that lower read count/cell will decrease the barcode recovery, still as an alternative it is possible to recover the cell transcriptome and barcode by 5’ 10X kit or Vasa seq[(7)](https://paperpile.com/c/6cJReo/MbNg).

9) One thing that is interesting to check in the data is the existence of inactive and active HSC clones based on their output, similar to a recent previous report (ref. 17 in the manuscript). Just skimming the barcode data in Figure 3, it looks like some of these inactive HSCs are also seen but not followed up at all. It would be interesting to check whether the current study, using now busulfan instead of irradiation, reaches similar conclusions regarding active/inactive HSC clones and their signatures. Also, it seems interesting that when fewer clones engraft (mouse 4), this behavior of active/inactive clones is no longer apparent, suggesting that only when a sufficiently diverse pool of HSCs engraft can these differences in HSC behavior arise. **It would be interesting to compare the transcriptional signatures of HSCs activated by Plt depletion (Fig. 4-5 of present manuscript) with those from ref 17.**

We have compared signatures of Plt activated clones with clones from [(8)](https://paperpile.com/c/6cJReo/6c6N) activated upon serial transplantation, we have observed a limited overlap of 0.6% (Figure 5F, full list of genes with fold change expression in Supplementary Table 8). This low overlap may result from different biological cues leading to HSC activation (platelet depletion or transplantation), reflecting diverse signalling pathways being involved, different library construction methods, read depth and analysis pipelines. In the current study we have used Seurat package, followed by Deseq2 analysis for differentially expressed genes in pseudobulk samples (3 pseudobulk samples generated by adding gene expression matrices of single cells belonging to the active or inactive clone type based on their PB output in one animal). That is a different strategy compared to summing all single cells of active or inactive behaviour as routinely performed in scanpy analysis and also in Reference [(8)](https://paperpile.com/c/6cJReo/6c6N).

10)  Another uniquely addressable question is, for instance, **why are some lineages so much more abundant in the BM than in the circulating Plt/RBC pool.** In fact, are HSC clones that produce circulating Plt transcriptionally different from those that produce just BM MkPs/CFU-E but fewer circulating cells? There seems to be a few examples of both types of clones in the dataset shown (at least in mouse 2 and mouse 3).

We have performed the analysis to compare LT-HSC clones producing only progenitors with those contributing to progenitors and mature cells. Results of this analysis are included in Figure 3J, main text page 8. Full list of DE genes is included in Supplementary Table 5.

Observed gene expression signature suggests that LT-HSC producing only progenitors are more quiescent, which correlates with a downregulation of *Klf1* and *Nupr1*. Furthermore, downregulation of *Ccr1* implies inhibition of differentiation towards mature myeloid cells.

The other unique superiority of the technique is obviously shown in Figure 5, where **dynamic shifts of clonal behaviours are observed through time within the same**

**mouse, and then compared with transcriptomes of HSCs**. Yet again, this dynamic capacity to sample all blood lineages from the PB within the same mouse (**which I argue is the most important aspect of this technology, directly linked to the most novel and impactful results of this work, in figure 5**) is somehow not the central argument given in the main text or the discussion.

We have highlighted the novelty of our approach in the edited manuscript title, abstract and introduction following the suggestion from Reviewer1.

11) "Cells from each type clustered together, regardless of experimental group, indicating that the changes in PB clonality is not reflected by a global shift in progenitor gene expression". I don't think this wording is correct. There could have been a shift in gene expression during the initial response (before the 10 days post-challenge), which was just not measured, because the researchers allowed the system to restore almost to the steady-state. **What is actually being observed is that there is no global transcriptional memory of the challenge and thus the challenged and non-challenged cells are essentially indistinguishable by unsupervised clustering**. The results from supervised clustering, equally, reveal that LT-HSCs that switched to platelet production during stress actually did retain a bit of a transcriptional memory of the challenge (even if globally the cell state is unaltered). This is actually one of the most interesting and novel aspects of this study, and

it's really a pity that this wasn't also assayed after a longer period of time (say 30 days), which would better ensure that this is a proper transcriptional memory of the platelet-depletion.

We agree with the Reviewer 1 and we have adjusted the wording regarding the transcriptional memory on page 10.

12) I suspect that Figure 5f and 5g are mislabeled. Grey should be before plt depletion and orange should be after. This should be clarified in the legend as well. In fact, in general, the legends for figures 3-4-5 do not contain the necessary information to understand these plots.

That is correct, we apologise for mislabelling the legend; it has been corrected in the revised version of the manuscript, Figure 4F and G.

13) Several figures and legends have other problems too and they might all benefit from a little sprucing up. For instance: some plot legends are too small to read; most scales and bars are not appropriately explained; the meaning of certain symbols in plots (such as ** in figure 5d) is not explained in legends and, if referred to statistical tests, there are not detailed in the legend either; some figure panels are not referenced in the main text (Fig 6b-c).

We have increased font size in our figures, annotated statistical tests being used. We have updated the text to reference the figures.

**Reviewer #2:**

In this manuscript, the authors used RNA barcoding to study the blood production of individual hematopoietic stem cell clones after transplantation in mice. The authors claimed "for the first time including anucleate and nucleate lineages", but this is incorrect. A couple of published studies have already recovered barcodes from erythrocytes and platelets, including the one that was referenced (Reference #17). Most results presented in this manuscript validate known knowledge in the field. Probably the only new and interesting finding is that "acute platelet depletion shifts the output of multipotent HSCs to the exclusive production of platelets". However, the data presented did not directly and sufficiently show this point, nor the underlying mechanism. For example:

We thank Reviewer #2 for their thoughtful and detailed comments. We have done our best to address the raised questions and our point by point responses follow.

**What fraction of multipotent HSC clones become exclusively producing platelets?**

We have observed a switch in the blood output from multipotent (PEMB) to exclusively platelet (P, PEB, PE) producing clones in 40-85% (depending on the animal, Figure 4F, main text page 9.

**What are their differentiation and gene expression characteristics comparing to those multipotent HSC clones that do not exhibit this shift?**

Following this suggestion we have removed the panel showing GEO of DE genes between PEMB clones in vehicle treated animals and PE clones in platelet depleted animals, since here we were comparing clones in different animals. We have followed Reviewer's 2 suggestion to compare PEMB and P/PE clones in the same animal (LT-HSC clones experienced exactly the same treatment).

We have performed differential expression analysis between P/PE clones and PEMB clones after the platelet depletion. We have detected 3670 differentially expressed genes (Supplementary Table 6) and performed GEO analysis showing enrichment for DNA replication and DNA repair genes (Figure 5B, full list included in Supplementary Table 6) .

**There was only one data point analyzed after the platelet depletion. It would be interesting to see the dynamic changes in response to the platelet depletion in time course.**

Thank you for this comment, we have performed a new experiment in which we have monitored the clonal kinetics up to 12 weeks post platelet depletion. In brief recipients treated with busulfan were transplanted with 2,800 LT-HSC; blood samples were collected at 12, 28 weeks, 14 days before platelet depletion (depicted as bas-base line in Figure 6B-F). Next, we have evaluated clonal composition in PB at 10 days, 4-, 8-, and 12 weeks post platelet depletion along with isolation of bone marrow populations including: GFP+ LT-HSC, GFP+ MkP and GFP+ CFU-E using FACS. BM populations were sorted in bulk and used only for clonal analysis. Samples were processed as described previously. Performed analysis has revealed again clonal switching after platelet depletion. These results are included in Figure 6A-F and in Supplementary Figure 7.

Major:

1. 	Figure 1b is not sufficient to demonstrate the correlation of barcode recovery from cDNA and from gDNA. **It is important to compare individual barcodes, instead of the total read of all barcodes as in this figure**.

 We agree with the Reviewer 2 and we have plotted normalized read count from gDNA and cDNA obtained from 3 independent clones:

To evaluate the correlation between cDNA and gDNA retrieved reads we have run the generalized linear model, adjusted R2 = 0.729 indicates a strong correlation between cDNA and gDNA retrieved read number.

Above plot together with the linear regression model is included in Supplementary Figure 1A-B and in the main text, page 4.

2. 	"We reliably detected barcodes in as few as 1 cell (Figure 1) and clones that represent only 0.01% of the starting population were successfully detected, while they were missed at DNA level (Figure 1B)." (Page 3). **These claims are not supported by the cited figures**.

We agree with this comment and to address this question in a separate experiment we have tested the size of the smallest clone, which we can detect in the unequally mixed samples using cDNA or gDNA, results have been added to Figure 1C. As shown in this figure we can reliably detect the smallest clone contributing  0.089% and the 2nd and 3rd smallest at 0.89% using cDNA, however not detecting it with gDNA. gDNA most likely requires higher sequencing depth, thus cDNA is more cost efficient and universal molecule for barcode detection in single BM stem and progenitor cells and their anucleate cell progeny in PB.

Based on Figure 3A in the manuscript we are confident that we can detect barcodes in a single cell with over 90% efficiency.

3. 	**It is unclear why this study only considers barcodes that collectively account for >90% total abundance** (Page 4).

It has been shown that cellular barcoding is reliable at detecting medium size and large clones, however it struggles with detection of small clones, because they are indistinguishable from sequencing noise. Thus, there have been several approaches to overcome this limitation of the method, some groups apply various tools depending on the structure of the barcode for semi random barcodes such as Shannon count [(9–11)](https://paperpile.com/c/6cJReo/2aLCT+QdDGF+NDHKn) or top 90% of present barcodes identified in the sample using saturation curve [(12)](https://paperpile.com/c/6cJReo/xafzj), others apply the exact match to the constant fragment of the PCR handle downstream the barcode and recover a barcode sequence (by design random 17 nucleotides)  allowing up to 4 mutations within the barcode [(5,13–15)](https://paperpile.com/c/6cJReo/K7Evr+n3IMh+BC2UV+UvPqi). In this manuscript we have applied a saturation curve to define a set of dominant barcodes accounting for >90% of total abundance and call ‘rare’ the remaining clones. We then focused our clonal dynamic analyses on dominant barcodes, which robustly contribute to hematopoiesis.

This comment has now been included in the Methods section, page 35.

4. 	"Fluctuations of clone size could be observed with some variation at week 12 and barcode composition becoming more similar among lineages at later time points (Fig. 1B, Suppl. Fig. 3A-D)." (Page 5) These claims are not supported by the cited figures.

Apologies for the inconsistency between the text and the figures.

The text should have read:

Fluctuations of clone size could be observed with some variation at week 12 and barcode composition becoming more similar among lineages at later time points (Fig. 2F-I). This has been corrected, page 6.

5. 	Fig. 2F-I does not support the claim "These analyses demonstrate that clonal composition within cells of the same lineage is stable over time." (Page 5). For example, in Mouse 1, wk 12 M is closer to wk 28 E than to wk 28 M; wk 28 B is closer to wk 12 E than to wk 12 B; wk 28 E is closer to wk 12 M than to wk 12 E.

We agree with the Reviewer2 and appreciate this comment. We have replotted our data scaling both axes to reflect the contribution of Dim1 and Dim2, updated Figure 2 F-I.

We observe high similarity of barcodes in the same lineages at different timepoints, although as pointed out some time points especially in case of Myeloid lineage are more variable and have a longer distance between each other (Figure 2F-I). It may relate to lower RNA content in myeloid cells. All cell types except for Platelets are relatively consistent on Dim1 over time. Platelet variation might be related to the amount of RNA, which is 10 000 times lower compared to nucleated cells.  Increased stochasticity in myeloid cells may have originated from limited sampling at an early time point and low RNA yield in this cell type due to their low transcriptional activity [(16)](https://paperpile.com/c/6cJReo/WTo1). This comment has been included in the main text, page 5.

6. 	Figure 4G is missing but discussed in the result section: "This group of barcodes was also found in erythroid cells and B cells (Figure 4G, platelet-erythroid-Bcell clone, PEB clones), which can be explained by the half life time of the latter two cell types (22 days and 13-22 weeks respectively [31,32], strongly suggesting they have been produced before the platelet depletion, the time when the clone was multipotent and supported all analyzed blood lineages (Figure 4F, G)." (Page 8)

Apologies, the text should have read:

This group of clones was also found in erythroid cells and B cells (Figure 5G, PEB-platelet-erythroid-B cell clones), which can be explained by the half life time of the latter two cell types (22 days and 13-22 weeks respectively [31,32], strongly suggesting they have been produced before the platelet depletion, the time when the clone was multipotent and supported all analyzed blood lineages.

Information relevant for this text is now in Figure 4F. The text  has been edited in the manuscript, page 9)

7. 	Figure 5b, it is unclear why the differential gene expression analysis was performed by comparing "multipotent clones in control animals with P/PE/PEB clones in platelet depleted animals". (Page 8). Have the authors compared (1) multipotent clones between control animals and platelet depleted animal?

We apologise for being unclear. Yes, in the initial submission we have compared multipotent clones in control/vehicle treated animals and P/PE/PEB clones in animals which underwent platelet depletion.

Following the Reviewer’s 2 suggestions we have compared P/PE/PEB clones with PEMB clones in platelet depleted animals.

We have updated figure 5B accordingly. Full list of GEO terms is included in Supplementary Table 6.

As suggested by Reviewer2 we have also performed differential gene expression analysis between multipotent clones in control and PEMB clones in platelet-depleted animals and have detected only 1 differentially expressed gene-*S1pr3*, which was upregulated in multipotent clones in platelet-depleted animals. S1pr3 is a receptor for the bioactive lipid sphingosine-1-phosphate, is a central regulator which drives myeloid differentiation and activates inflammatory programs in both HSC and LSC [(17)](https://paperpile.com/c/6cJReo/4NjM), suggesting that HSC clones in platelet depleted animals are actively contributing to myelopoiesis to replenish platelets and myeloid progeny. This information is not included in the main text.

8. 	Figure 5E, what is the definition of repurposed multipotent clones (PEB clones)?

Multipotent clones (PEMB), which underwent repurposing, had to contribute over 0.089% into all lineages at 2 time points before platelet depletion and <0.089% to myeloid cells upon platelet depletion. This comment has been included in the main text, page 9.

9. 	Figure 5G is not discussed anywhere.

It has now been included in the revised version (page 9, as Figure 4G).

Minor:

1. 	"Relative measurement error of barcode detection at DNA level was 13%, while for cDNA was 16%." (Page 3). It is unclear how the "relative measurement error" is calculated.

It has been included in Methods section, page 37:

2. 	Experimental detail is missing about the technical replicates in Suppl. Fig. 1f-i. Were the replicated samples processed separately starting from gDNA or cDNA isolation?

This information was added to Supplementary Figure legend 2. For this correlation we have used samples from mice 1-4.

3. 	Figure 2b-e, does the same color represent the same barcode in each graph? This needs to be clarified in the figure legend.

No, the colours were reused between panels. We apologise for not being clear. This information has been added to the Figure 2 legend.

4. 	Figure 3e-h and Figure 4f, what does the color show? What does the color key unit "scale" mean? How is "spearmen correlation" showed in these figures? These need to be clarified in the figure legend.

We have added the explanation of how the correlation has been calculated in legends for Figure 3 and 4.

5. 	In figure 4b, wk28# E data dot is missing from the graph.

This has been corrected in updated version, Figure 4b.

6. 	For scRNA seq analysis (Figure 5), were the cells harvested at week 28? How many cells of each type were harvested? How is the data quality?

Yes, cells were harvested at week 28. Exact cell numbers in all 3 populations are included in Supplementary Table 3, QC is included in Supplementary figure 4B-E.

7. 	Figure 5B, Log10 (FDR) color is difficult to read from the graph. Also, the full list of enriched GO terms needs to be provided in supplemental material.

We removed this figure and substituted it with suggested by Reviewer2 DE genes between PE and PEMB clones in platelet depleted animals rather than having this comparison between PEMB clones in vehicle treated animals (Figure 3E-H) and PE clones in platelet depleted ones (Figure 4H).

1.	[Mistry, J. J. *et al.* ROS-mediated PI3K activation drives mitochondrial transfer from stromal cells to hematopoietic stem cells in response to infection. *Proc. Natl. Acad. Sci. U. S. A.* **116,** 24610–24619 (2019).](http://paperpile.com/b/6cJReo/1ljo4)

2.	[Marlein, C. R. *et al.* CD38-Driven Mitochondrial Trafficking Promotes Bioenergetic Plasticity in Multiple Myeloma. *Cancer Res.* **79,** 2285–2297 (2019).](http://paperpile.com/b/6cJReo/6H9E1)

3.	[Moore, J. A. *et al.* LC3-associated phagocytosis in bone marrow macrophages suppresses acute myeloid leukemia progression through STING activation. *Journal of Clinical Investigation* **132,** Preprint at https://doi.org/](http://paperpile.com/b/6cJReo/iLzu5)[10.1172/jci153157](http://dx.doi.org/10.1172/jci153157) [(2022)](http://paperpile.com/b/6cJReo/iLzu5)

4.	[Hellmich, C. *et al.* p16INK4A dependent senescence in the bone marrow niche drives age-related metabolic changes of hematopoietic progenitors. *Blood Adv* (2022). doi:](http://paperpile.com/b/6cJReo/RZ1NL)[10.1182/bloodadvances.2022007033](http://dx.doi.org/10.1182/bloodadvances.2022007033)

5.	[Fan, X. *et al.* Clonal tracking of erythropoiesis in rhesus macaques. *Haematologica* **105,** 1813–1824 (2020).](http://paperpile.com/b/6cJReo/UvPqi)

6.	[Replogle, J. M. *et al.* Combinatorial single-cell CRISPR screens by direct guide RNA capture and targeted sequencing. *Nat. Biotechnol.* **38,** 954–961 (2020).](http://paperpile.com/b/6cJReo/cGv2b)

7.	[Salmen, F. *et al.* High-throughput total RNA sequencing in single cells using VASA-seq. *Nat. Biotechnol.* (2022). doi:](http://paperpile.com/b/6cJReo/MbNg)[10.1038/s41587-022-01361-8](http://dx.doi.org/10.1038/s41587-022-01361-8)

8.	[Rodriguez-Fraticelli, A. E. *et al.* Single-cell lineage tracing unveils a role for TCF15 in haematopoiesis. *Nature* **583,** 585–589 (2020).](http://paperpile.com/b/6cJReo/6c6N)

9.	[Belderbos, M., de Haan, G., Koster, T., van der Velden, V. & Bystrykh, L. Subclonal evolution of pediatric acute lymphoblastic leukemia revealed by genetic barcoding. *Experimental Hematology* **44,** S45 Preprint at https://doi.org/](http://paperpile.com/b/6cJReo/2aLCT)[10.1016/j.exphem.2016.06.055](http://dx.doi.org/10.1016/j.exphem.2016.06.055) [(2016)](http://paperpile.com/b/6cJReo/2aLCT)

10.	[Belderbos, M. E. *et al.* Donor-to-Donor Heterogeneity in the Clonal Dynamics of Transplanted Human Cord Blood Stem Cells in Murine Xenografts. *Biol. Blood Marrow Transplant.* **26,** 16–25 (2020).](http://paperpile.com/b/6cJReo/QdDGF)

11.	[Jacobs, S. *et al.* Quantitative distribution of patient-derived leukemia clones in murine xenografts revealed by cellular barcodes. *Leukemia* **34,** 1669–1674 Preprint at https://doi.org/](http://paperpile.com/b/6cJReo/NDHKn)[10.1038/s41375-019-0695-2](http://dx.doi.org/10.1038/s41375-019-0695-2) [(2020)](http://paperpile.com/b/6cJReo/NDHKn)

12.	[Ferrari, S. *et al.* Efficient gene editing of human long-term hematopoietic stem cells validated by clonal tracking. *Nat. Biotechnol.* **38,** 1298–1308 (2020).](http://paperpile.com/b/6cJReo/xafzj)

13.	[Lu, R., Neff, N. F., Quake, S. R. & Weissman, I. L. Tracking single hematopoietic stem cells in vivo using high-throughput sequencing in conjunction with viral genetic barcoding. *Nat. Biotechnol.* **29,** 928–933 (2011).](http://paperpile.com/b/6cJReo/K7Evr)

14.	[Wu, C. *et al.* Clonal tracking of rhesus macaque hematopoiesis highlights a distinct lineage origin for natural killer cells. *Cell Stem Cell* **14,** 486–499 (2014).](http://paperpile.com/b/6cJReo/n3IMh)

15.	[Bramlett, C. *et al.* Clonal tracking using embedded viral barcoding and high-throughput sequencing. *Nat. Protoc.* **15,** 1436–1458 (2020).](http://paperpile.com/b/6cJReo/BC2UV)

16.	[Garratt, L. W. Current Understanding of the Neutrophil Transcriptome in Health and Disease. *Cells* **10,** (2021).](http://paperpile.com/b/6cJReo/WTo1)

17.	[Xie, S. Z. *et al.* Sphingosine-1-phosphate receptor 3 potentiates inflammatory programs in normal and leukemia stem cells to promote differentiation. *Blood Cancer Discov* **2,** 32–53 (2021).](http://paperpile.com/b/6cJReo/4NjM)

**Second round of review**

**Reviewer 1**

This revised version of the manuscript by Wojtowicz contains several improvements and new results that address most of my concerns. I think this will prove to be a very interesting new study, showcasing the possibilities of taking advantage of RNA barcode detection in anucleated cells to delineate shifting clonal dynamics in these complicated, yet essential, blood lineages, in a way that has not been done before.

**Reviewer 2**

I appreciate the efforts that the authors made to improve this manuscript. Unfortunately, I still don't think that there is enough technical or conceptual advance to justify publication at this stage. On the technical side, barcode analysis of the anucleate cells is not novel. The authors did not respond to this novelty issue, and still claim "In summary, our novel barcoding approach enables true multi-lineage kinetic analysis of hematopoietic output in both anucleate and nucleate lineages for the first time.", which is inappropriate. Regarding the conceptual advance, both reviewers highlighted the finding that "acute platelet depletion shifts the output of multipotent HSCs to the exclusive production of platelets". However, the data presented still don't demonstrate this point adequately or provide any mechanistic insights. For example, Figure 4F is not a good way to show what fraction of HSC clones exhibit this shift. It is unclear how often this shift occurs and how important the shifted clones are in generating the platelet. Moreover, it is unclear what is the short term and long-term significance of this shift. The Figure 5 gene expression analysis does not reveal any mechanistic insights. Figure 6b-f is confusing, as it is unclear what each line represents. Furthermore, a very concerning problem of this manuscript is that there is no statistical significance calculated in any of the barcode analyses. None of the conclusions are supported by statistics. This is surprising considering the quantitative data that the barcode analyses generate. Below are a few comments regarding specific figures.

1. New Supplementary Figure 1, the figure legend for A and B seems reversed. The correlation shown in B is not great. One would expect much better correlation based on other published studies. This raised concerns about the data quality in this manuscript.

2. New Figure 1C y-axis is difficult to read. From this plot, it is difficult to compare the clonal abundance similarity of the same barcodes between different samples, as some barcodes, especially the abundant ones, are difficult to see. Scatter plots could be a better choice.

3. I am still not convinced that Fig. 2F-I show "high similarity of barcodes in the same lineages at different timepoints". The authors reasoned that "some of the early time points are more variable". But there are only two time points in this comparison, so the "variable early time points" basically means the comparison between different time points are not accurate.

4. In response to the main point #6, the author stated "This group of clones was also found in erythroid cells and B cells (Figure 5G, PEB-plateleterythroid-B cell clones)," There is no Figure 5G.

**Authors Response**

**Point-by-point responses to the reviewers’ comments:**

We are grateful to Reviewer 1 for the positive overall assessment of our study and their thoughtful and constructively critical comments. We have responded to each point in a comprehensive manner, as detailed in the point by point reply below. The most important novel data shown in the revised manuscript are summarised below:

1.We have discussed previous RNA clonal analysis performed in RBCs by the Dunbar group (1) in the introduction and discussion. Furthermore, we have highlighted 2 completely different mechanisms of biogenesis of platelets and RBCs further reinforcing the novelty of our findings in the platelet lineage.

2.We have carefully compared cDNA and gDNA (Suppl. Figure 1B) barcode retrieval correlation and compared with the recent study (2). In revising this that we observed that this previous publication used the Pearson correlation coefficient to evaluate barcode composition in cDNA and gDNA samples whereas, to our disadvantage, we had used a generalised linear model. After calculating Pearson correlation using the data in Supplementary Figure 1B the Pearson correlation in our study was r=0.85, in fact higher than in the above paper (r=0.72).

3.To simplify plotting of the panel in Figure 1C we have followed the suggestion and presented data points as a scatter plot with connecting line and standard deviation in the updated Figure 1C. Statistical significance was calculated using 2 way ANOVA and obtained p values are provided in Supplementary Table 1A. For the inspection of both replicates we have provided a scatter plot with all replicates included as separate dots in Suppl. Figure 1C.

4.Following Reviewer 1’s suggestion we have presented data as a Pearson correlation coefficient between early (week 12) and late (week 28) timepoints in updated Figure 2F-I, Figure 4B-E. As shown, correlations between later time points are higher compared to the early ones. We have provided Pearson r correlation coefficients and p values in the Supplementary Table 3.

5.To summarise and statistically compare clonal composition and output after the platelet depletion we have performed a 2 way ANOVA with Bonferroni multiple comparisons test correction (updated Figure 4F).

6.To simplify the analysis of results obtained in the long-term follow up study we have calculated the Pearson correlation coefficient (Figure 6B-D) and summary of clone type and output before the platelet depletion, 10 days and 12 weeks after the perturbation (Figure 6E). Due to high variability in this experiment we have observed trends similar to those observed in Figure 4F, however this did not reach the statistical significance.

1.Fan, X. et al. Clonal tracking of erythropoiesis in rhesus macaques. Haematologica 105, 1813–1824 (2020). 2.Rodriguez-Fraticelli, A. E. et al. Single-cell lineage tracing unveils a role for TCF15 in haematopoiesis. Nature 583, 585–589 (2020).

**Third round of review**

**Reviewer 1**

This new revision of Wojtowicz et al contains a lot of updated analyses that address most of my previous concerns. I really enjoyed reading some of the detailed comments and updates on conclusions. A lot to unpack from these findings, which I am sure will influence the field for a long time. Notably, the authors have improved statistical analyses throughout the manuscript, addressing past concerns of both reviewers. In my opinion, they have done tremendous work. In sum, the technical breakthrough and the observations reported here, while some being preliminary, are greatly useful to the community of researchers and I still hold my view that they very-well pass the standards of peer review.

One specific comments: I would remove statements such as "for the first time" or "the first successful" in the abstract and main text. First, because these age poorly when time passes. But also, because some people might disagree on details or take it too personal. There is no need for these types of claims, for which objectivity is hard to assess, and also they add little to the scientific solidity of the article.
